# Stretchable, Twisted Conductive Microtubules for Wearable Computing, Robotics, Electronics, and Healthcare

**DOI:** 10.1038/s41598-017-01898-8

**Published:** 2017-05-11

**Authors:** Thanh Nho Do, Yon Visell

**Affiliations:** Department of Electrical Computer Engineering, Media Arts and Technology Program, California NanoSystems Institute, University of California, Santa Barbara, CA 93106 USA

## Abstract

Stretchable and flexible multifunctional electronic components, including sensors and actuators, have received increasing attention in robotics, electronics, wearable, and healthcare applications. Despite advances, it has remained challenging to design analogs of many electronic components to be highly stretchable, to be efficient to fabricate, and to provide control over electronic performance. Here, we describe highly elastic sensors and interconnects formed from thin, twisted conductive microtubules. These devices consist of twisted assemblies of thin, highly stretchable (>400%) elastomer tubules filled with liquid conductor (eutectic gallium indium, EGaIn), and fabricated using a simple roller coating process. As we demonstrate, these devices can operate as multimodal sensors for strain, rotation, contact force, or contact location. We also show that, through twisting, it is possible to control their mechanical performance and electronic sensitivity. In extensive experiments, we have evaluated the capabilities of these devices, and have prototyped an array of applications in several domains of stretchable and wearable electronics. These devices provide a novel, low cost solution for high performance stretchable electronics with broad applications in industry, healthcare, and consumer electronics, to emerging product categories of high potential economic and societal significance.

## Introduction

An increasing array of soft electronic devices have been investigated as alternatives to conventional planar, brittle, and rigid electronics, in order to facilitate emerging applications that can benefit from the high levels of flexibility and stretchability that are made possible via new material, fabrication, and design methods. The unique mechanical properties of soft electronic devices will allow future integrated electronic systems to seamlessly conform to curved or variable surfaces, including the human body, leading to new applications in areas including wearable computing, surgical robotics, field robotics, manufacturing, entertainment, and rehabilitation. However, due to the intrinsic properties of these materials and processes, considerable research is needed if we are to realize functional devices that can approach the levels of performance, multifunctionality, and integration that are attainable with conventional electronic design methods.

Electronic devices can be broadly described as mechanically rigid (non-flexible), flexible, or stretchable. Advances in materials and in fabrication technologies have led to new platforms and the commercial proliferation of flexible sensors and other devices with low bending stiffness, but very low stretchability, due to the high bulk elastic modulus of the substrates^1–3^. The engineering of soft, stretchable electronics, remains more challenging, due to the paucity of electronic fabrication techniques, and the high bulk modulus of most electronic materials. Emerging categories of soft, precise mechanical strain sensors hold promise for robotics, wearable computing, and healthcare applications^4–9^, and the development of skin-wearable soft mechanical (strain and stress) sensors has also attracted considerable attention^10–21^. To enable practical applications of soft and stretchable electronic devices, improvements in electrical and mechanical robustness are needed^22–24^.

The possibility of realizing stretchable, conductive polymer fibers has attracted interest because these materials can be designed to be highly deformable, cost effective, and adaptable to requirements that arise in areas such as wearable computing. The most common methods for integrating conductive materials into soft polymer filaments include coating with metal or carbon^21, 25^, or with conductive composites of polymer with graphite, carbon, metal nanoparticles, or other conductive materials^13, 15, 23, 24, 26–29^. This can yield devices that are useful as strain (stretch) sensors, but that may not admit other functionalities (such as contact sensing, as demonstrated here). Many of the aforementioned sensors, and some multifunctional devices proposed in the literature, utilize nanostructured materials, such as graphene, carbon nanotubes, or metal nanomaterials, in order to realize their functionality, sometimes at the cost of added manufacturing complexity^23, 30, 31^. Existing techniques are not able to realize monolithic sensors that can perform multiple modes of mechanical sensing while attaining high levels of stretchability such as we demonstrate. Consequently, conventional electronic sensors for bending, rotation, and force sensing are not able to match the mechanical and functional properties of the devices presented here (Table S1, Supplementary Information), which are, as we demonstrate, more readily adaptable to the human body or other soft structures.

In this paper, we report new methods for the design of highly stretchable electronic devices, based on structures that we refer to as *twisted conductive microtubules*, which prove multifunctional, low in cost, and facile to fabricate. As we demonstrate, these structures can operate as sensors for strain or rotation, with electronic performance that can be tuned dynamically, after manufacture. We also show that these devices can sense contact forces, and that, when woven into mesh structures, they can sense contact over a distributed area. The same devices are also able to serve as highly conductive electronic interconnects. We report on the performance of these sensors, highlighting their fast electronic response, high levels of durability, and very high stretchability. In order to illustrate the large range of potential uses of these devices, we also demonstrate several applications, including wearable movement sensing, contact force sensing for wearable computing, position and rotation sensing in cable-driven robotics, tactile sensing, and electronic power interconnects. Together, their attributes make the devices suitable for numerous applications in soft electronics, robotics, healthcare, industry, and wearable computing.

We also introduce a new fabrication method in order to realize these twisted microtubules, based on a simple roller coating process that is easy to realize using standard tooling, and that can even be reproduced using a hand operated electric drill, as we show. This method is simple but highly effective, amenable to fabrication in research lab settings or to being adapted for large scale manufacture leading to cost-effective commercialization. The sensor makes use of a liquid metal alloy conductor (eutectic gallium indium, EGaIn) that is injected into the hollow channel of the microtubules. Upon twisting pairs of the microtubules, the deformation of the sensor is continuously altered, and, by controlling the number of twisted turns, we demonstrate that it is possible to greatly shape the mechanical and electronic performance of the sensor. The resulting twisted microtubules are able to achieve high sensitivity, dynamic range, and fast response times, while simultaneously being highly stretchable and durable. They can be used in stretchable interconnects and stress or strain conductive sensors that are suitable for numerous applications in soft electronics, robotics, healthcare, and wearable computing.

## Results and Discussion

### Design and Fabrication of Twisted Conductive Microtubules

**Figure 1:**
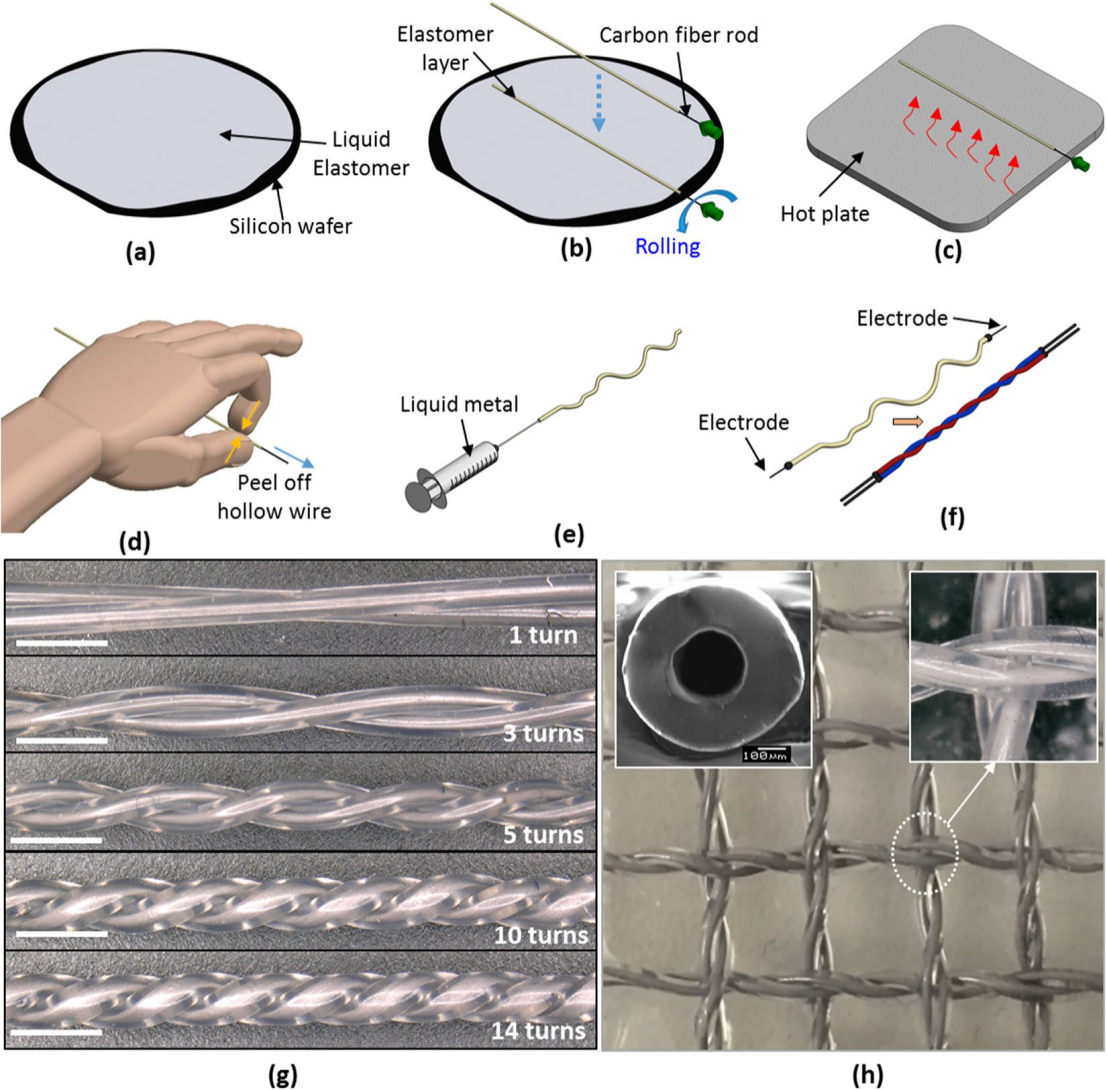
Fabrication process for the conductive microtubules. (**a**) Spin coat a thin layer of liquid polymer (here, Ecoflex 00-30) onto a silicon wafer, (**b**) rolling coat the carbon rod with the thin layer of liquid polymer, (**c**) heat with a hot plate, (**d**) peel off the hollow microtubule, (**e**) inject liquid metal (EGaIn) into the hollow microtubule using a needle and syringe, (**f**) insert electrodes into both ends of the microtubules and twist. Structure of the microtubules with (**g**) different numbers of turns per twisted pair, (**h**) woven electronic sensing fabric using twisted conductive microtubules. Detail: Cross-sectional SEM image of microtubule. Bar scale: 100 µm.

The twisted microtubule sensors are based on soft polymer tubules that are filled with liquid metal alloy conductor (eutectic gallium indium, EGaIn) and formed into pairs of intertwined (twisted) channels (Fig. 1). When stretched, the resistance of a microtubule pair increases predictably and monotonically. As explained and demonstrated below, the strain sensitivity, or rate of increase in resistance with elongation, increases monotonically with the quantity of twisting that is applied, facilitating a simple method for tuning sensor performance.

Figure 1(a–f) illustrate the fabrication process. Briefly, a hollow silicone microtubule is fabricated using a simple roller coating process. Addition cured soft polymer (Ecoflex 00-30, Smooth-On, Inc., USA) is mixed with a weight ratio 1:1 (part A: part B) and spun onto a silicon wafer of desired thickness. Then, a fine carbon fiber rod is rolled onto the wafer, and subsequently heated by a hot plate. Because the polymer has a high elongation at failure, the thin walled tubule that results is easily peeled off the carbon rod. Different rod diameters can be selected in order to control the inner diameter of the hollow microtubules. The thickness, and hence the outer diameter, is controlled by varying the number of laminated layers. Figure 1(h) shows the cross section of the conductive microtubule using a 200 µm carbon fiber rod with three laminated layers of polymer. A conductive microtubule is obtained by injecting liquid metal alloy (EGaIn, 75% Ga, 25% In by mass, melting point 15.7 °C) into the hollow channel via needle and syringe, and terminating the end on electrodes. Other conductors, such as ionic saline solutions, could also be used.

To produce strain sensors, the microtubules are folded, forming pairs of conductive paths, and are twisted, intertwining the halves of the tube (Fig. 1(g) and Fig. S3). As demonstrated in our experiments and from basic mechanical and electrical considerations, reviewed below, the electronic sensitivity increases with the quantity of twisting that is applied. The same microtubule structures may also be used, with or without twisting, as soft electronic interconnects (Fig. 1(e,f)), opening further applications in wearable computing and robotics, and may be combined into mesh structures that can perform distributed tactile sensing (See Fig. 1(h)).

The fabrication method offers several advantages. It is low in cost, and is time efficient, technically simple and easy to reproduce. With it, one can realize soft conductive channels with inner diameters that are small (as low as 0.2 mm in our prototypes), limited only by the diameter of the carbon rods (CST, Composite Store Inc., CA). The thickness of the microtubules is controlled by varying the number of rolling elastomer layers. Fig. S2 presents SEM images of cross sections for different sizes of microtubules.

In addition, this method is able to realize complex structures, in the form of twisted, intertwined channels, that are prohibitively difficult to realize using other fabrication methods, including existing MEMS and soft lithography techniques. Mechanically, the resulting conductive microtubules possess high levels of stretchability (>400% in our prototypes), resulting in devices that are highly flexible and compliant. As discussed below, they also make it possible to realize sensors with improved, programmable, sensitivity.

### Mechanical and Electrical Performance: Linear Strain and Rotation

**Figure 2:**
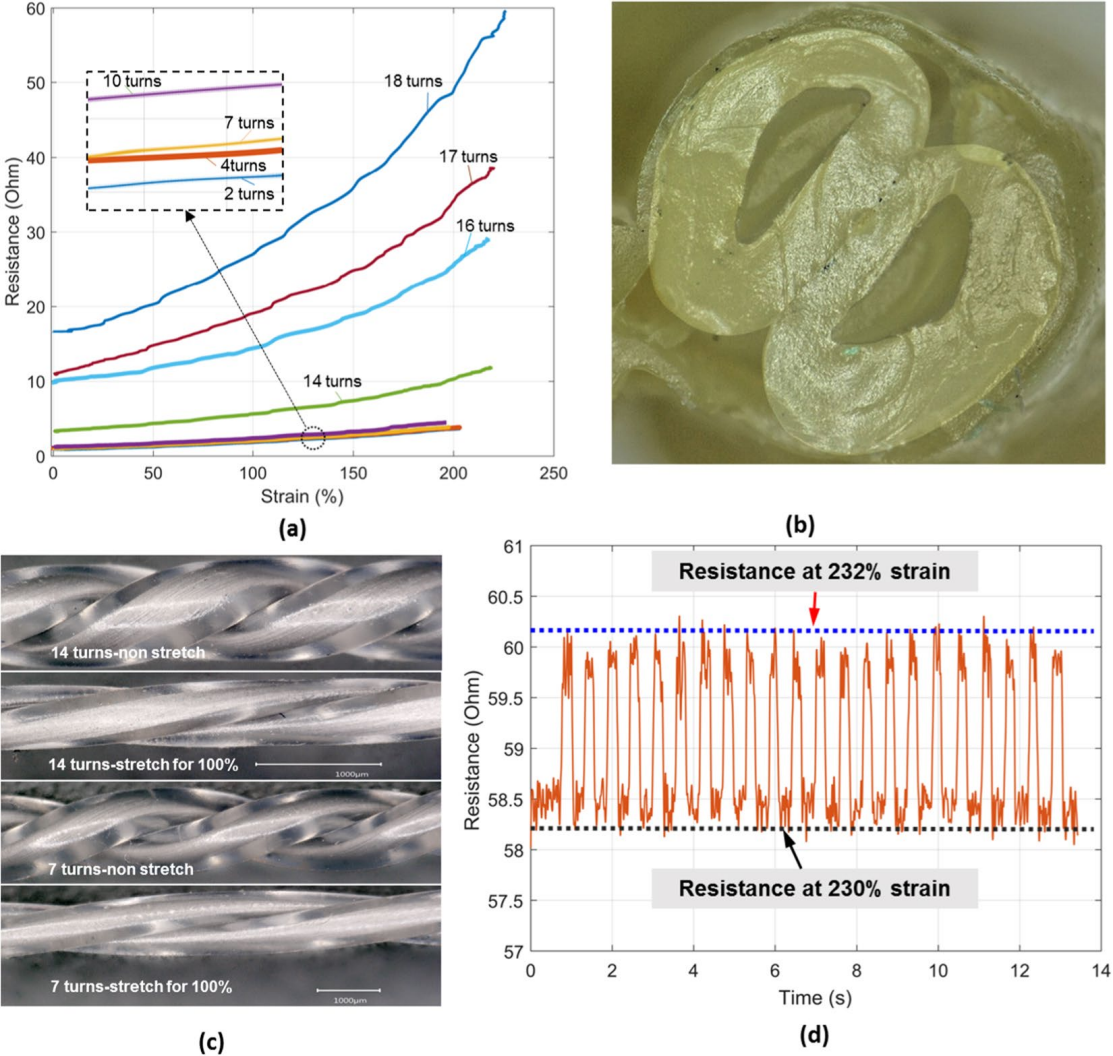
Characterizing the mechanical and electrical performance of the twisted conductive microtubules. (**a**) Strain versus electrical resistance curve for microtubules with different amounts of twisting. (**b**) Optical image of the deformed cross section of a twisted microtubule pair (6 turns, 100% strain). (**c**) Optical images of the twisted microtubules with different turns in non-stretch (0%) and 100% strain conditions. (**d**) Change in resistance for twisted microtubule (18 turns, 230% nominal strain) under low amplitude (2%) cycling loading, illustrating the high sensitivity.

In order to characterize the strain sensing performance of these twisted conductive microtubules, we tested several samples that were loaded in the axial direction (See Fig. 1(g)). As shown in Fig. 2, we observed an increase in resistance with increasing strain. In tandem with this, the cross section was changed due to stretching and compression.

The resistance of a microtubule depends on the resistivity of the liquid metal (ρ), cross sectional area *S*, and its length *L*, as described by Pouillet’s Law. When stretched, the end to end resistance *R* increases due to the change in length and area of the conductor, *R* = *ρLS* ^*−*1^, where *ρ* is the resistivity, *L* is the length. EGaIn is highly conductive, with *ρ* ≈ 2.9 × 10^−6^ Ω cm. The conductor is nearly incompressible, and its volume *V* is constant. Thus *S* = *VL* ^*−*1^, so that *R* = *ρL* ^2^ *V* ^*−*1^, where *V* is volume, a constant. This quadratic dependence of resistance on length is reflected in our measurements (Fig. 2).

For the same reasons, an increase in resistance is expected to accompany an increase in the number of rotations of a twisted channel pair. Twisting increases the nominal length *L* of the channel, yielding, by the argument above, a growth in resistance, *R* = *ρL* ^2^ *V* ^−1^. The sensitivity *dR/dL* is thus predicted to grow monotonically with *L*. We observed such a growth in resistance with twisting in our measurements (Fig. 2(a)). Using a device with nominal untwisted length *L*_0_ = 40 mm, we applied strains from 0 to 200%. Both the resistance and the rate of increase in resistance with strain monotonically increased for quantities of turns greater than about ten, n ≥ 10 (Fig. 2(a), inset), indicating that the sensitivity of the device increased with additional twisting. This can be explained by the decrease in cross sectional area and increase in length with twisting of the microtubules. The shape of the inner channel also deformed with twisting, attaining a non-disc, “D” shape (See Fig. 2(b)). The inner channels with greater numbers of turns were deformed more than that twisted microtubules with fewer number of turns. Figure 2(c) shows an optical image of twisted microtubules with 14 turns and 7 turns at a nominal strain of 100% (Fig. S3 provides additional data). At high levels of twisting, the sensor is able to detect very small changes in strain, with changes of just 2% strain at 230% total strain readily apparent in resistance measurements obtained from a twisted (18 turn) sensor (Fig. 2(d)).

**Figure 3:**
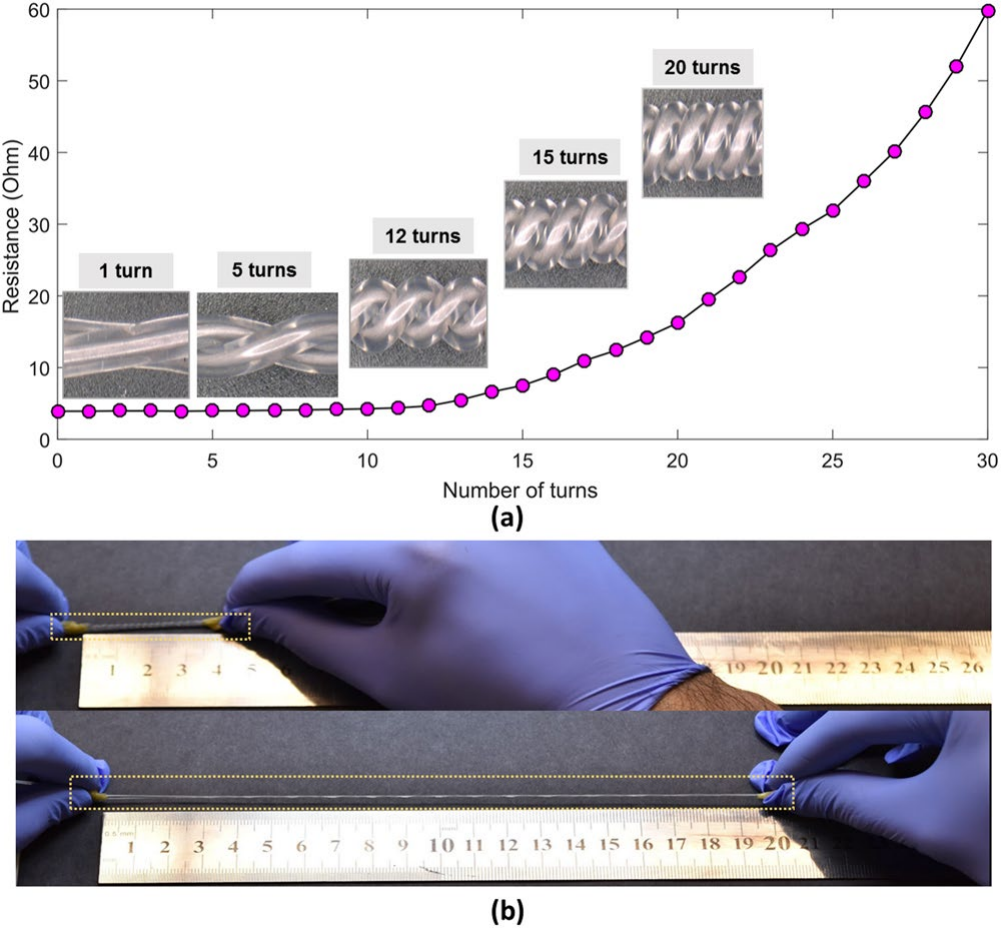
Mechanical and electrical performance. (**a**) Beyond 12 turns, resistance rises monotonically with additional rotation. (**b**) Image showing 100% and 600% strain (14 turns). Nominal length was 40 mm in both experiments.

The devices also afford another mode of sensing, as their resistance increases monotonically when twisted, for the same reason noted above, allowing them to function as rotary encoders. For a twisted microtubule pair with a rest length of 40 mm, and subjected to a twisting pre-load of 13 turns, one readily observes a monotonic increase in resistance with rotation (Fig. 3(a)). There is little precedent for rotational sensing in soft, filament-like sensors. As discussed below, this proposed mode of operation has interesting implications for robotic systems based on twisted string actuators.

**Figure 4:**
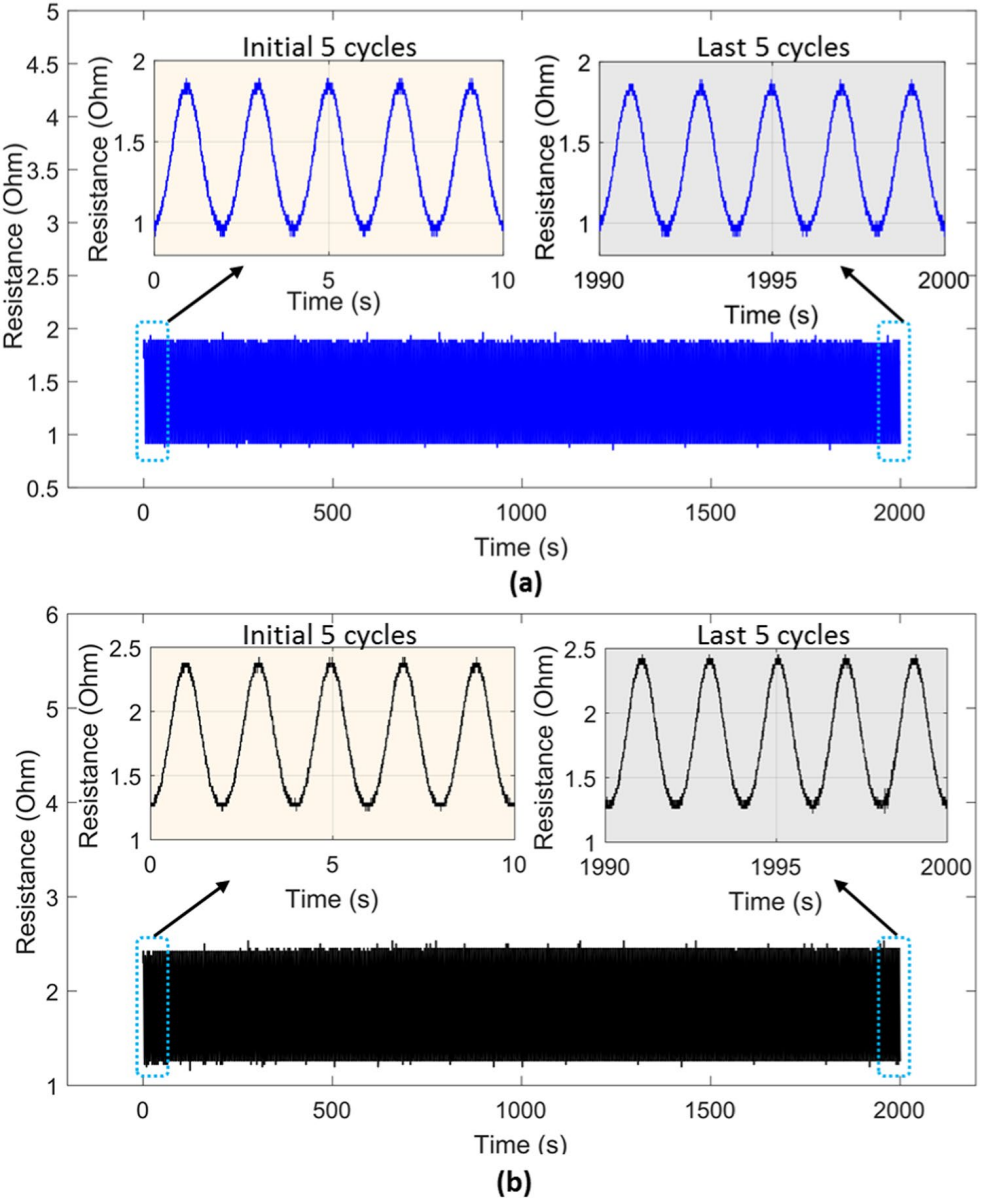
Evaluating the stability of the sensor output during repeated loading to 100% strain, over 1000 cycles (period: 2 seconds). (**a**) Resistance vs. time, 4 turns (**b**) Resistance vs. time, 10 turns. Insets (both Figures): Detail view of signal during the first 5 cycles and the last 5 cycles.

The twisted microtubules exhibit high strength and deformability before failure. They may readily be stretched beyond 600% without damage (Fig. 3(b)), and remain electronically functional up to a failure elongation of 750%. The devices also exhibit high durability Fig. 4(a,b)), with the sensor output closely following cyclic loading for 1000 or more cycles. The results indicate that the electronic performance is stable and reproducible under high strains and cycles, and that performance is unchanged between the first and last cycles (Fig. 4, inset; Further experimental details: Fig. S8).

### Contact Sensing

**Figure 5:**
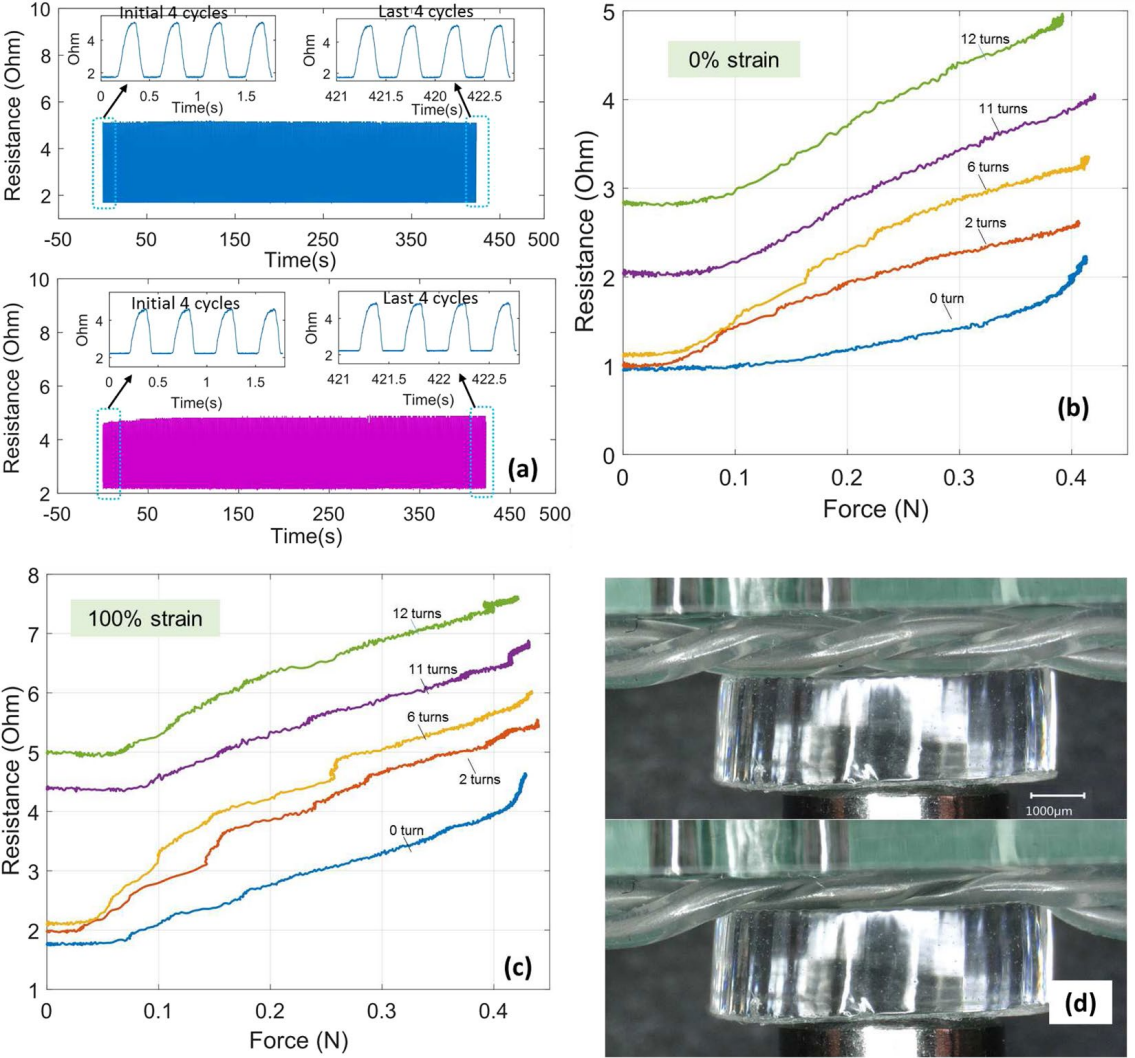
Contact sensing for different device configurations (twisting, pre-load, and strain), (**a**) When cyclic loading is applied (period 0.7 seconds), sensors respond consistently under repeated loading over several hundred cycles. Top: 6 turns. Bottom panel: 11 turns. (**b**) Resistance increased with force applied to an unstretched sensor (0% strain) at all levels of twisting, and (**c**) likewise increased with force applied to a stretched sensor (100% strain). (**d**) Images showing the deformation of the twisted microtubules with applied pressure (6 turns shown). The nominal untwisted length of the tested devices was 40 mm. Other details were as described in the main text.

Pressing on a microtubule squeezes the channels, eliciting large and rapid changes in the sensor output (Fig. 5(b and c)), and yielding an increase in resistance that is monotonic with applied force. The resistance *r* per unit length of the channel is given by *r* = *ρS* ^*−*1^, where the (approximately elliptical) cross section area is *S* = *π x y*, and *x* is the semi-normal axis in the direction of loading, while y is the complementary axis. Small contact forces *F* yield a small displacements *F* = *kΔx*, where *k* is effective channel stiffness, yielding localized changes in resistance per unit length *r* = *ρ* (*π* [*x*_0_ + *Δx*] *y*) ^*−*1^ = *r*_0_ (1 + Δ*x*/*x*_0_). Thus, resistance grows with the relative normal load applied to the microtubule. The rate of change is independent of the longitudinal strain, since it depends only on the ratio Δ*x*/*x*_0_. A constant offset in resistance is also produced, due to the net lengthening of the channel. This description is consistent with findings in Fig. 5(c), where the slope of contact force with resistance can be observed to be approximately independent of the degree of twisting. At very low strain conditions (zero twisting, 0% strain, as in the bottom curve of Fig. 5(b)), different behavior is observed. We hypothesize that this is because the low strain alters the wall response mechanics. A more detailed mechanical account of this behavior will require a geometric and solid mechanical analysis of the helical geometry of the tubular pair under normal stress loading at low longitudinal strains. This analysis is beyond the scope of the current paper, but one we plan to address in future work.

To experimentally characterize the contact sensitivity, a force tip of 3 mm, mounted on a load cell (see Fig. S7) indented the sensor. Little change in resistance was observed for low levels of applied force (below about 80 mN), during a period of the initial deformation of the microtubule structure (Fig. 5(c)). Although the compression of the twisted microtubule structure depended on the sensor geometry (Fig. 5(d)), the results proved highly repeatable and dynamically stable under repeated loading, spanning hundred cycles (period 0.7 seconds), irrespective of the amount of twisting (Fig. 5(a)). The results demonstrate that the sensor output is sensitive and robust during contact (force) sensing.

## Applications

### Human Motion Sensing

**Figure 6:**
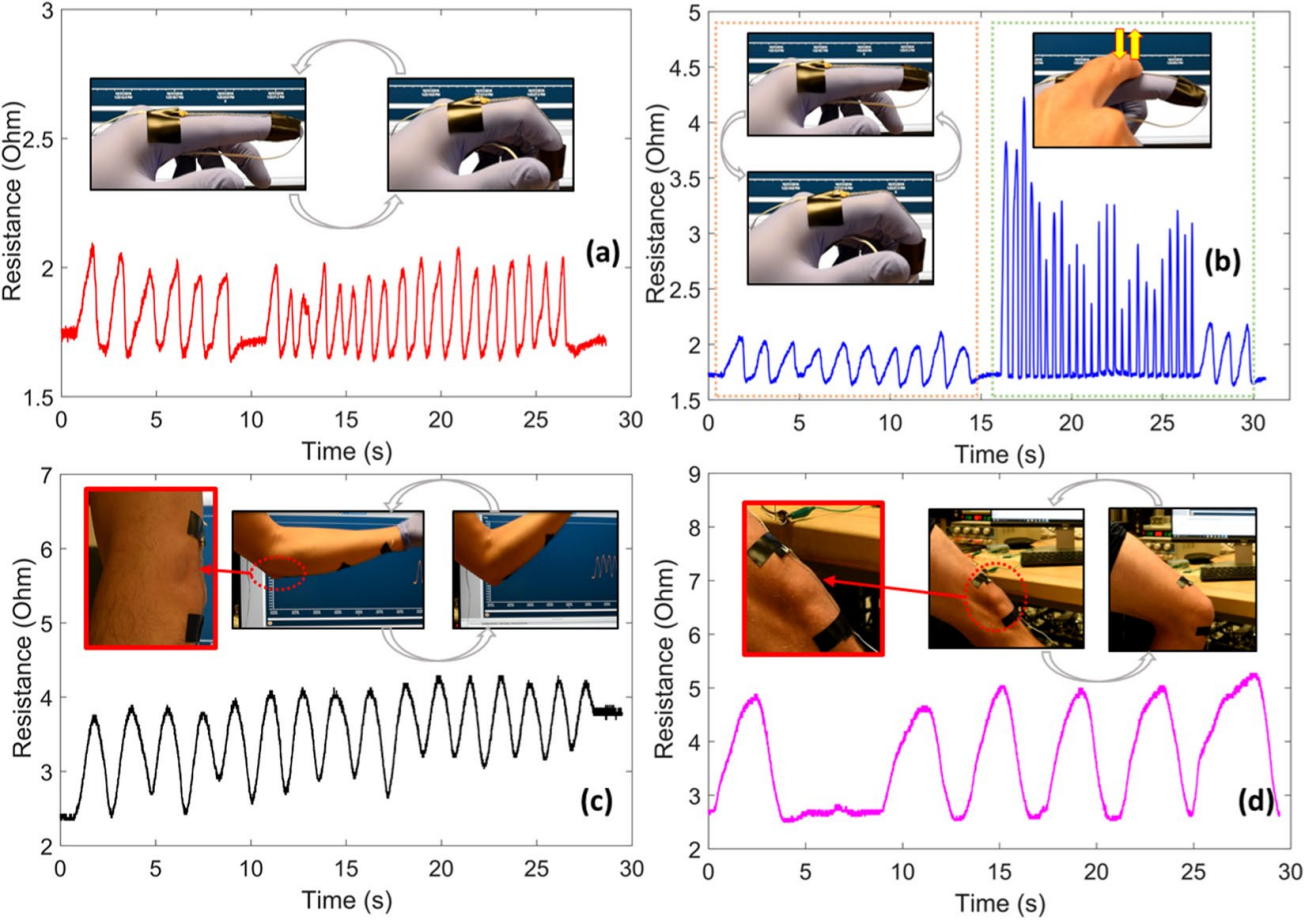
Sensing motion via twisted conductive microtubules. (**a**) Capturing bending motion near the human proximal interphalangeal (PIP) joint, (**b**) Sensing motion and contact with the same device: motion is sensed in the first 15 seconds, force in the next 15 seconds, and motion in the last three seconds. (**c**) Capturing motion at the elbow (**d**) and knee (**c**). Signals were recorded in real time.

The stretchability, compact size, sensitivity, robustness, and multimodal functionality of these sensors make them suited to a range of wearable sensing tasks, including sensing the motion of small or large joints in the body. In experiments, we demonstrated the ability of the sensor to capture bending of a human finger (Fig. 6(a)) when attached near the joint via adhesive tape. Bending causes the inner channels of twisted conductive microtubules to be deformed due to the strain and compression of the sensor, yielding a recoverable increase in resistance with bending angle. The same device can also sense contact to the finger (Fig. 6(b)), and if desired respond to both at once. In Fig. 6(b), motion is sensed in the first 15 seconds, force in the next 15 seconds, and motion in the last three seconds. The same device is readily adapted to sensing motion at larger joints, such as the human elbow (Fig. 6(c)), including movements more than 90°, and knee (Fig. 6(d)) The twisted microtubules are able to consistently detect motion over several cycles, with resistance increasing with bending angle.

Long-term monitoring of human joint motion plays an important role in many applications, especially in medicine and rehabilitation. These results indicate that these twisted microtubules can be used for detecting motion of the upper or lower limb. Their small size and low cost make them excellent candidates for applications in this field^12^. (Further examples are presented in Fig. S9 to S14).

### Motion Sensing for Cable-Driven Robotic Systems

**Figure 7:**
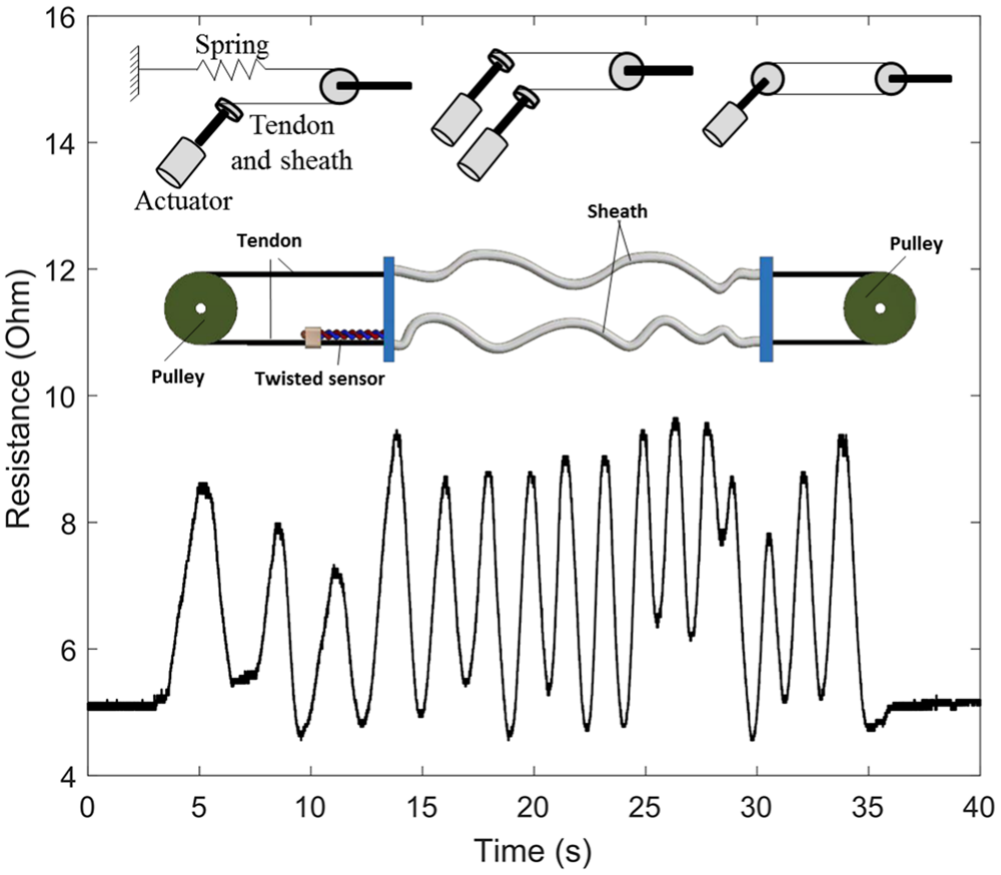
Sensing displacement in a tendon-driven robotic system. Bottom: Measurements (recorded in real time) demonstrating position sensing in a system configured as shown above, integrating twisted conductive microtubule sensor (10 turns). Top: Three prototypical configurations for a tendon-driven robotic system.

The aforementioned cases, involving body motion capture, also evoke another area of potential application for twisted microtubule sensors: in cable- or tendon-driven robotic systems. Figure 7 illustrates three prototypical configurations, in which the sensors are used to provide position feedback to a servo controlled robotic system. To demonstrate the practical feasibility of such an arrangement, we configured a pair of tendon-sheath-pulley mechanisms with position feedback from a microtubule strain sensor, one end of which was attached at the sheath fixture, while the other was connected to the tendon (Fig. 7, insets). Motion of the tendon strains the twisted microtubule sensor, evoking a commensurate change in resistance (Fig. 7), indicating the potential utility of these sensors in tendon drive systems, which are widely used in surgical, rehabilitation, and industrial applications^6, 32–34^.

Another potential robotic application of these devices would integrate them in a twisted string actuator system, a promising candidate for future robotic drive systems^35^. In such a system, the twisted microtubule sensors could be employed to sense twisting, because resistance increases with the quantity of turns (Fig. 3(a)), or to sense displacement (Fig. 7). The ability to sense rotational motion may also be applied to other rotational actuators and devices where a soft, small or dimensionally compact size is required.

### Contact Sensing in Surgery, Wearable Electronics, Healthcare, and Robotics

**Figure 8:**
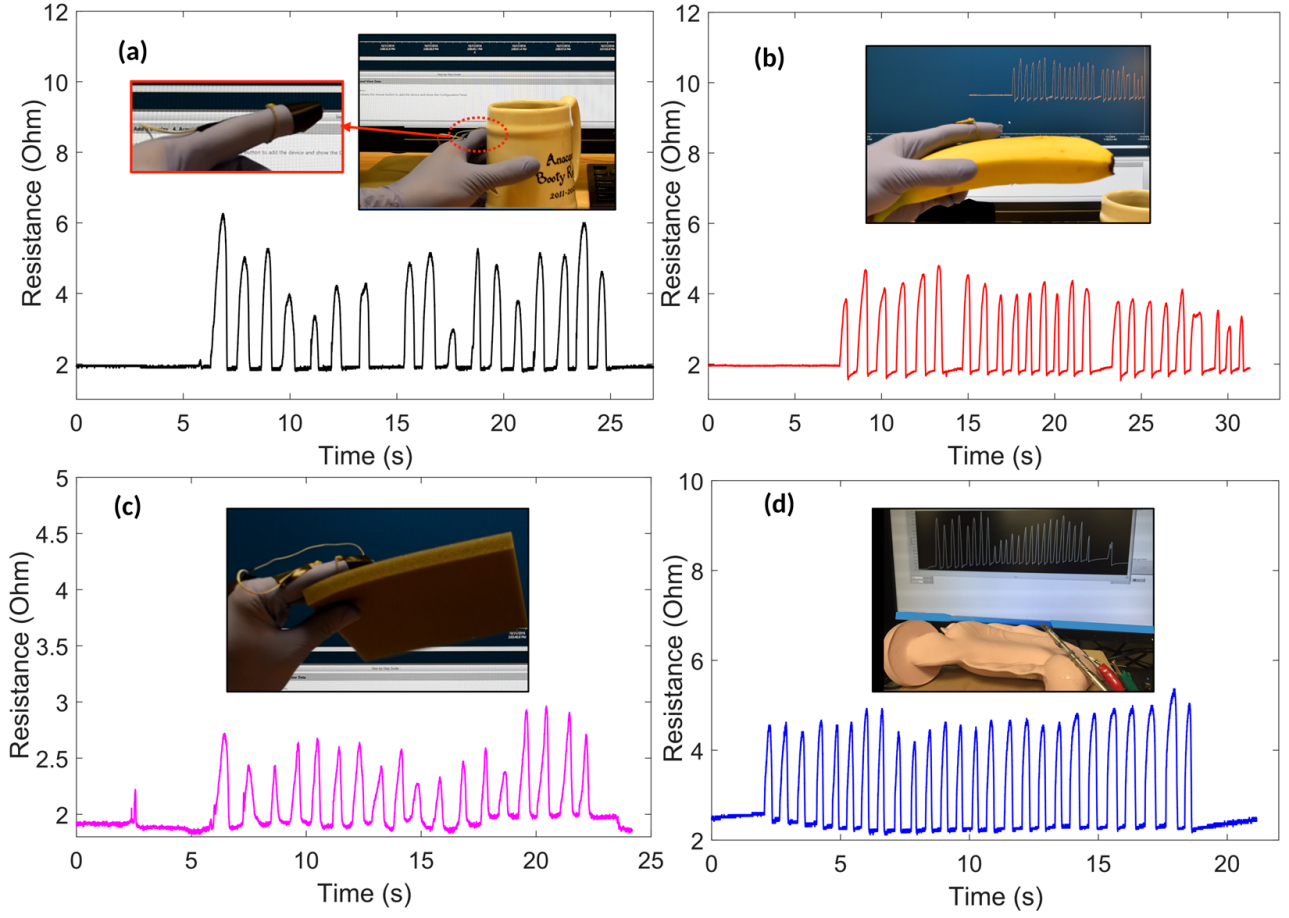
Contact sensors based on twisted microtubule structures is readily integrated in an array of applications. (**a**) Sensing forces as a human hand grasps a cup, (**b**) grasps a banana, or (**c**) grasps soft foam. (**d**) Sensing contact forces between surgical forceps and simulated tissue.

These sensors also prove useful for contact force sensing in wearable electronics, surgery, healthcare, and robotics. Their simple design makes it straightforward to adhere them to the body, or to attach them to a mechanism or tool, using standard adhesive tape or prosthetic glue. When a sensor is routed along the contact surface of a human or robot finger, it may readily be used to capture interaction forces during object grasping (Fig. 8(a–c)). The resistance changes rapidly with changes in the contact force between the surfaces of the limb and the object. Recorded contact signals prove stable and repeatable, growing ratiometrically with the forces involved when, for example, grasping a soft or hard object.

During surgical operations performed with a tool, there is a compelling need to measure interaction forces, in order to limit damage to tissues or to provide feedback to a human or robotic system. To demonstrate the adaptability of twisted microtubule sensors to such tasks, we integrated them with surgical forceps (Model A63010S, Olympus Corporation, Japan). The results (Fig. 8(d)) indicate that the sensors are able to provide a clear force signal reflecting interactions between the forceps and simulated tissue (Virtual Stomach, Chamberlain Group, USA), reinforcing their utility for applications in medicine, and for similar problems in healthcare and industry.

### Woven Twisted Microtubules as Electronic Skin for Tactile Sensing

**Figure 9:**
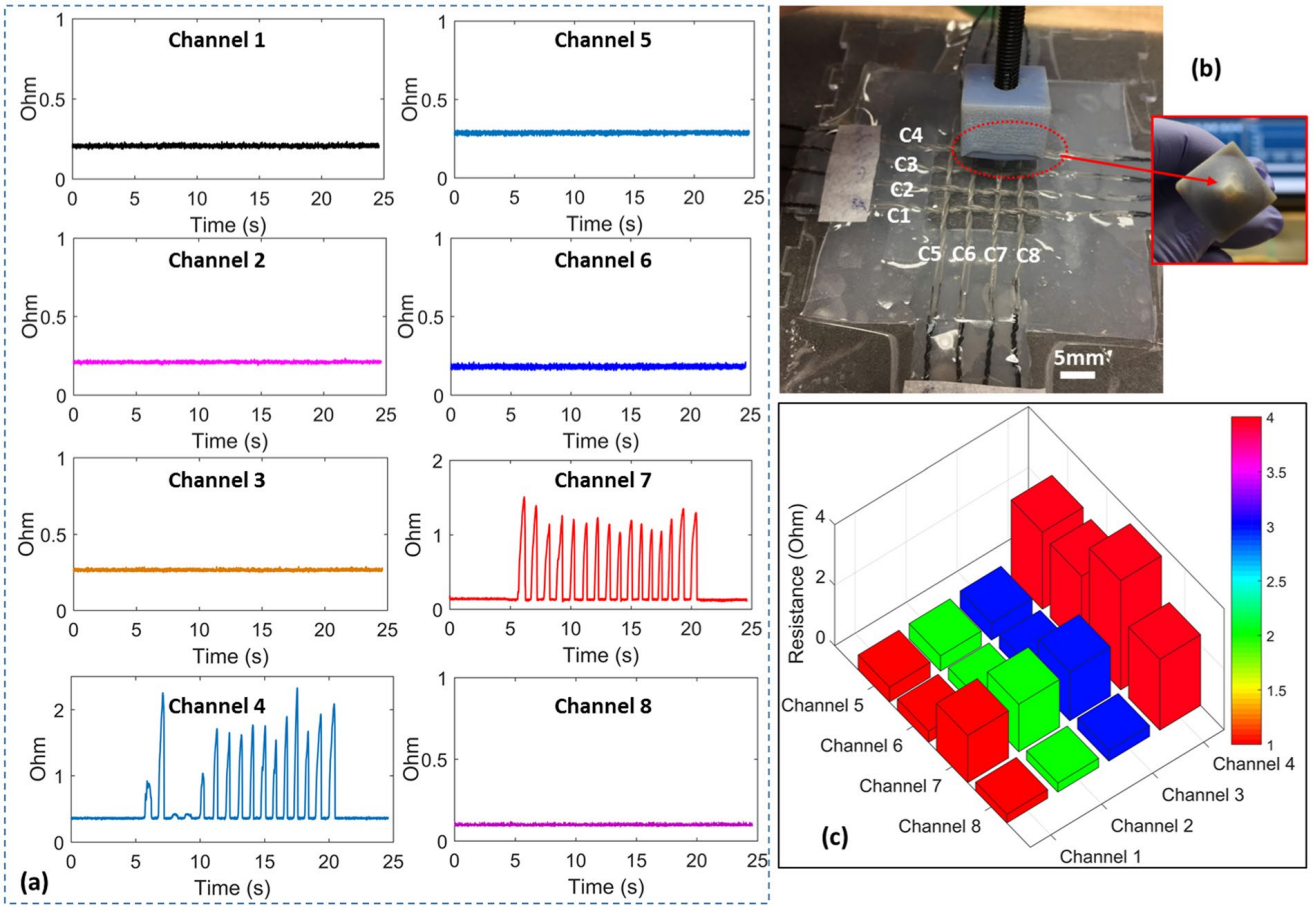
Tactile sensing with a woven array (eight channels, 4 × 4 cells) of twisted conductive microtubules. Tip displacement: 3 mm. (**a**) Force sensing in each of the eight channels, (**b**) Optical image of the array. (**c**) Tactile data illustrating interaction with the array. The peak signal correctly localizes the indentation to the cell at the intersection of channels 4 and 7.

The fiber-like quality of these structures makes it possible to weave them into two dimensional structures for distributed sensing applications, yielding electronic fabric that retains remarkably high levels of stretchability, electrical conductivity, and sensitivity. To illustrate these possibilities, we fabricated a woven array (eight channels, 4 × 4 cells, 5 mm spacing) of microtubules in which each intersection forms a single sensing cell (Fig. 9). Using the array, it is possible to measure time-varying contact forces (Fig. 9(a)) and to localize them within the domain of the sensor (Fig. 9(c)). Further contact examples are given in Fig. S16 in the supporting information.

### Stretchable Electronics

**Figure 10:**
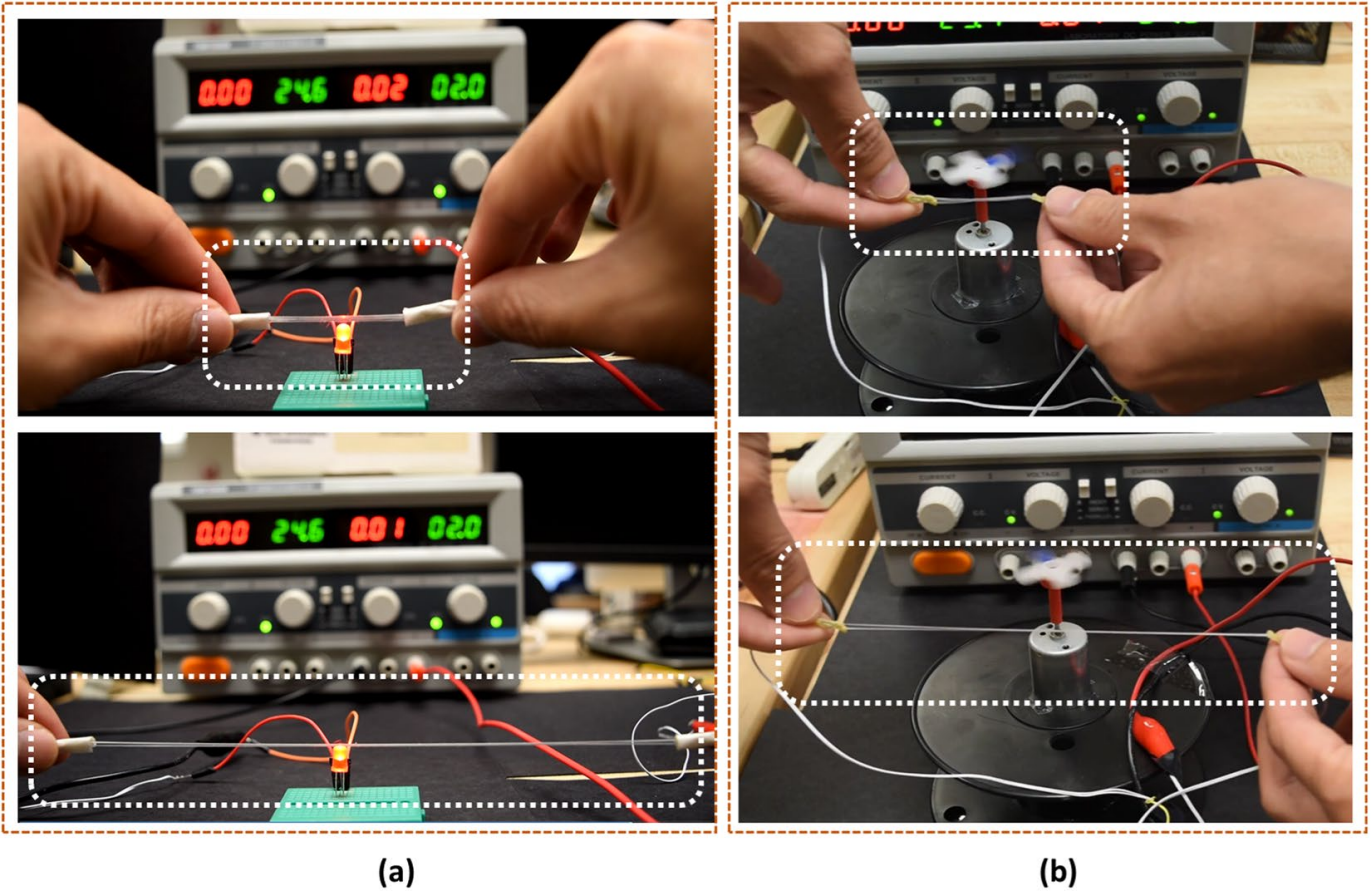
Untwisted microtubules, alone or in pairs (shown), can function as highly stretchable electrical interconnects. (**a**) Integrated in a functional circuit powering a LED. (**b**) Powering a rotary DC motor and blade.

In addition to their utility as sensors, the conductive microtubules can also serve as highly stretchable electronic interconnects, a topic that has attracted considerable recent interest^15, 23, 36, 37^. To illustrate these possibilities, we designed simple circuits in which electrical current flows from a DC power supply through an untwisted conductive microtubule, illuminating a light-emitting diode (LED; Fig. 10(a)) or supplying a rotary DC motor (Fig. 10(b)). The illumination of the LED and speed of the motor remain approximately constant even as the microtubule is stretched to greater than 400% strain, due to the low resistance (around 4 Ω) of the untwisted microtubule interconnect. These results confirm the utility of conductive microtubules as highly stretchable electronic interconnects. Further results are illustrated in the Supplementary Information (Supplementary Information, Fig. S1–S17, Table S1, and Movie S1–S15﻿).

## Conclusion

In summary, we have demonstrated highly stretchable conductive microtubules formed from thin, twisted (and non-twisted) conductive microtubules and a liquid metal alloy conductor (eutectic gallium indium, EGaIn). We presented a facile fabrication process based on roller coating, yielding cost-effective and scalable methods for the development of functional soft electronic devices.

These devices prove capable of sensing via multiple modalities: strain, contact force, rotation, and tactile sensing. Through an intertwining and twisting process, we demonstrate that it is possible control the rate of change in resistance during sensing by varying the number of turns with predictable effects, and also show that this makes it possible to tune the electronic sensitivity of the sensors in strain or rotation sensing. Compared to the commercial conventional sensors like motion/force sensors, our twisted microtubules provide potential alternatives and higher performances in terms of stretchability, flexible integration, sharp bend, multifunctional capability, and soft structure. Detailed comparisons can refer to Table S1, supplementary information.

As demonstrated in our experiments, these twisted microtubule sensors prove mechanically durable, with performance virtually unchanged after thousands of large-strain loading cycles, and robust, with electronic failure occurring at around 750%. The strain limit could be increased further through the use of other high elongation polymer composites, such as elastomeric polysiloxane nanocomposite (Gelest ExSil 100, elongation ≥5000%^38^).

The sensors are amenable to a wide range of potential uses, and we demonstrated several in this paper. These include wearable sensing of motion of the human body, contact force and tactile sensing for wearable computing, translation and rotation sensing in cable-driven robotic systems or twisted string actuators, tactile sensing of tool-tissue or hand-object interactions, and power transmission in soft electronics. Together, the attributes of these twisted microtubule devices make them suitable for numerous emerging applications in soft electronics, robotics, healthcare, industry, and wearable computing, pointing toward the large potential economic impact of this work.

## Methods

### Fabrication process for twisted conductive microtubules

First, liquid Ecoflex-0030 (Smooth-On, Inc, Easton, PA, USA) is prepared by mixing part A and part B with a weight ratio of 1:1. After mixing, the liquid solution is degassed in a vacuum chamber (Model VD 23, BINDER, Germany) for about 5 minutes to remove bubbles. Next, the silicone solution is deposited on a silicon wafer via spin coating (Spin Coater Model EDC-650-8B from Laurell Technologies Corporation, PA, USA). Subsequently, a fine carbon fiber rod with an outer diameter of 200 μm (CST- The Composites Store, Inc., CA, USA) is mounted onto an electric drill, which is used to roll the onto the thin layer of liquid silicone polymer. Next, the rod and silicone laminate is heated via hot plate (Model HP88854100, The Lab Depot, Inc., USA) at 100 °C to cure the elastomer. The wall thickness of the silicone microtubule is controlled by varying the number of laminated layers, by repeating the aforementioned process. During the experiments, we used three laminated layers for the microtubules. Because the Ecoflex-0030 has a high elongation at break, the silicone layers can be easily peeled off from the carbon rod by hand yielding unfilled microtubules (Supplementary Information, Fig. S1 and Movie S1). Using a narrow gauge needle and syringe, we inject the liquid metal alloy conductor (eutectic gallium indium, EGaIn) into the hollow microtubule. Contact cement adhesive glue (DAP products, Inc., USA) is used to seal the electric wires in the silicone layer. Finally, the two conductive microtubules are intertwined and twisted around each other with the desired quantity of turns in order to form the stretchable twisted conductive microtubules (Fig. S5).

### Geometric (deformation) characterization

The electronic performance of the microtubule sensors can be attributed to two factors: elongation and change in cross section area. In order to further illustrate the latter, we cut a small length of single microtubule and carefully align it onto the surface of the thin layer Ecoflex-0030 using micro tweezers. Subsequently, both microtubule and its silicone layer are heated at 100 °C for 15 minutes using the hotplate (Model HP88854100, The Lab Depot, Inc., USA). SEM images for the cross section of the microtubule (Fig. 1(h) and Fig. S2) are obtained using a FEI Helios Nanolab 600 ion beam microscope (operating voltage at 2 kV and current 60 pA). Due to the challenges in measuring the 3D geometry while the structure is subjected to large elongation, we used contact cement adhesive glue (DAP products, Inc., USA) as a channel material, freezing the structure in place. First, the adhesive glue in liquid solution is injected into the hollow channels of the microtubules using a needle and syringe. Thereafter, the two microtubules were preloaded and twisted as specified. The twisted microtubules were then immersed in liquid adhesive glue, which was allowed to cure at room temperature for 5 hours. We avoid using heating (which would accelerate the curing time) to avoid unexpected deformations. The resulting samples were cut in section at a predetermined position and put into a 3D optical microscope (VHX-5000, Keyence Corp., Japan) to obtain cross section images (See Fig. 2(b)). The same 3D Microscope was used to characterize the stress and strain for twisted microtubules with different twisted turns (See Figs 5 and S6).

### Measurement and analysis

The resistance for stress and strain characterization of the twisted conductive microtubules were measured at DC and digitized using data acquisition hardware (USB 1408FS from MicroDAQ, ltd., USA). The measurement circuit is shown in Fig. S4. Contact force characterization was performed using a miniature load cell (FUTEK LSB200, Advanced Sensor Technology, Inc., USA) and a potentiometer (PTF, State Electronics Part Corp., USA) was used to measure displacement during the strain validation. All of the obtained signals are processed in the MATLAB (The MathWorks Inc., USA) computing environment. All experimental configurations are presented in the supporting information.

## Electronic supplementary material

Supplementary Information

Movie S1. Removal process for the elastic microtubule after roller coating process and heating

Movie S2. Stretchability for the twisted microtubules with 14 turns and 100% of strain

Movie S3. Shape deformation of the twisted microtubules with 6 turns

Movie S4. Motion and contact force sensing when worn on a human finger

Movie S5. Motion sensing for the elbow joint

Movie S6. Motion sensing for the knee joint

Movie S7. Position feedback for cable-driven system

Movie S8. Force sensing when grasping a cup

Movie S9. Force sensing when grasping a banana

Movie S10. Force sensing when grasping a soft foam

Movie S11. Force feedback in a surgical tool

Movie S12. Woven microtubule structure comprising a tactile sensing array (4x4 sensing cells)

Movie S13. Twisted microtubules as stretchable electrical interconnect powering an LED

Movie S14. Twisted microtubules as stretchable electrical interconnect powering a DC motor

Movie S15. Stretchability of the woven microtubule based tactile sensing array (4x4 sensing cells)
