# Supplementary material for: Stretchable, Twisted Conductive Microtubules for Wearable Computing, Robotics, Electronics, and Healthcare
Source: Sci Rep. 2017 May 11;7:1753. doi: 10.1038/s41598-017-01898-8 (PMC5431990; doi:10.1038/s41598-017-01898-8)
Supplement: Supplementary file 1 — Supplementary Information [file 41598_2017_1898_MOESM1_ESM.pdf]

Supplementary Information

Stretchable, Twisted Conductive Microtubules for Wearable Computing, Robotics, Electronics, and Healthcare

Thanh Nho Do and Yon Visell

Department of Electrical Computer Engineering, Media Arts and Technology Program, California NanoSystems Institute, University of California, Santa Barbara, CA 93106

E-mails: *yonvisell@ece.ucsb.edu and dothanhnho@engineering.ucsb.edu*

Supplementary figures

Figure S1. Removal process for the elastic microtubule after roller coating process and heating: (a) Carbon fiber rod with its outer layers of Ecoflex-00-30, (b-c) Using hand to remove the outer silicon shell, (d) The resulting elastic microtubule with its SEM image of cross section.

Figure S2. SEM images of the cross section for the microtubule for different inner diameters of the channel and different wall thicknesses.

Figure S3. Optical images for twisted conductive microtubules with different strain and twisting number (left panel at 0% strain and right panel at 100% strain). The images are recorded via 3D Microscope (Model VHX-5000, Keyence Corp., Japan).

Figure S4. Resistance measurements for the twisted conductive microtubules. We use a resistor of 10 Ω that directly connected to the twisted microtubules. Data acquisition hardware (USB 1408FS, MicroDAQ, ltd., USA) was connected to a Dell Desktop computer to digitize the signal. The power supply was set at 1 volt. Let V_s be the analog signal measured from both end of the sensor, the resistance R_s can be obtained as $R_s = 10V_s / (1-V_s)$.

Figure S5. Conductive microtubules are twisted to specified degrees using a two part pin vise (Starrett, USA). One part is fixed on the base and the other is connected to a rotary bearing. The number of turns of the twisted microtubules is controlled by the number of rotational cycles from the Starrett rotational part.

Figure S6. Strain measurement for the twisted microtubules, using a potentiometer (Model PTF, State Electronics Part Corp., USA), a DAQ module (USB 1408FS, MicroDAQ, ltd., USA), and a MISUMI linear slider. The signals are captured in software (MATLAB, The MathWorks, Inc., USA).

Figure S7. Experimental setup for stress-strain characterization

Figure S8. Strain (left panel) and stress (right panel) cycling tests for the twisted microtubules using a two-axis linear voice coil positioning stage (Model VCS15-050-LB-01-MC-2, H2W Technologies, Inc., USA).

Figure S9. Optical images, bending motion sensing of the human finger using twisted microtubules with 10 turns, (a) Finger in a straight position, (b) Finger in a bending position, (c) Twisted sensor is forced by another finger, (d) Twisted sensor is mounted on the dorsal surface of left hand under continuous loading from right hand.

Figure S10. Optical images: Bending motion sensing of the human elbow and knee joints using twisted microtubules with 10 turns, (a and b) Bending motion sensing for the elbow joint when the arm is in a straight position or bent position, respectively, (c and d) bending motion sensing for the knee joint when the leg is in straight position and bending position, respectively.

Figure S11. Experimental configuration for evaluating the twisted microtubules as position sensors integrated within a tendon-sheath mechanism driven pulley system.

Figure S12. Experimental configuration for a tactile sensing array (4x4 sensing cells) composed of woven microtubules.

Figure S13. Mechanical stretchability for a tactile sensing array (4x4 sensing cells) worn on a human arm.

Figure S14. Integration of twisted conductive microtubules into a medical bandage (upper panel) or surgical tool (lower panel)

Figure S15. Loading and unloading behavior of the twisted microtubules under strain testing with 4 twisted turns. (Upper panel) Resistance versus strain, (Lower panel) Time history for the strain (left y-axis) and resistance (right y-axis).

Figure S16. Woven microtubules in a tactile sensing array. (a) Contact force sensing for individual channels. (b) Optical image of tactile array (tip displacement: 10 mm). (c) The maximum force in the array correctly identifies the location at which force is applied.

Figure S17. Strain versus resistance testing for untwisted microtubules (0 turn)

Supplementary table

Table S1. Comparison between twisted microtubules and other conventional commercial sensors

Supplementary movies

Movie S1. Removal process for the elastic microtubule after roller coating process and heating

Movie S2. Stretchability for the twisted microtubules with 14 turns and 100% of strain

Movie S3. Shape deformation of the twisted microtubules with 6 turns

Movie S4. Motion and contact force sensing when worn on a human finger

Movie S5. Motion sensing for the elbow joint

Movie S6. Motion sensing for the knee joint

Movie S7. Position feedback for cable-driven system

Movie S8. Force sensing when grasping a cup

Movie S9. Force sensing when grasping a banana

Movie S10. Force sensing when grasping a soft foam

Movie S11. Force feedback in a surgical tool

Movie S12. Woven microtubule structure comprising a tactile sensing array (4x4 sensing cells)

Movie S13. Twisted microtubules as stretchable electrical interconnect powering an LED

Movie S14. Twisted microtubules as stretchable electrical interconnect powering a DC motor

Movie S15. Stretchability of the woven microtubule based tactile sensing array (4x4 sensing cells)

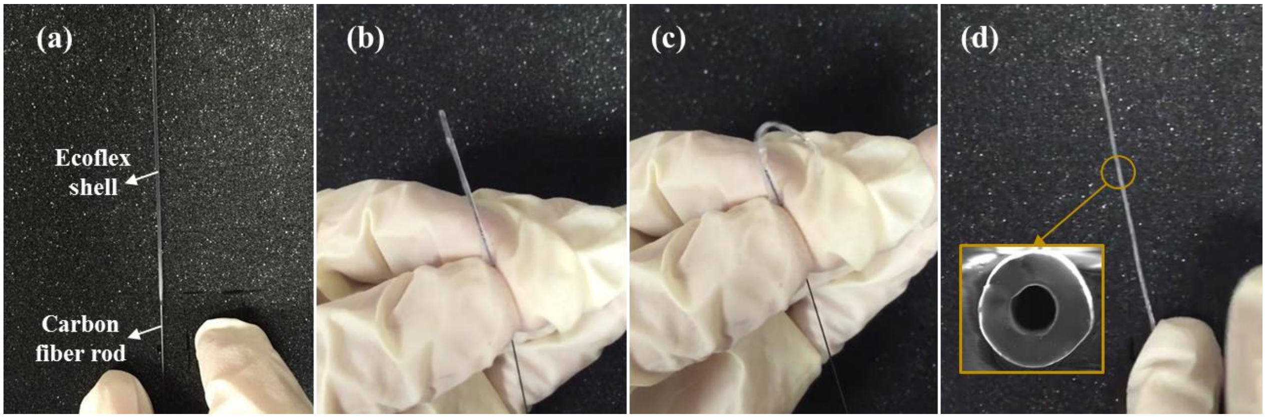

Figure S1. Removal process for the elastic microtubule after roller coating process and heating: (a) Carbon fiber rod with its outer layers of Ecoflex-00-30, (b-c) Using hand to remove the outer silicon shell, (d) The obtained elastic microtubule with its SEM image of cross section.

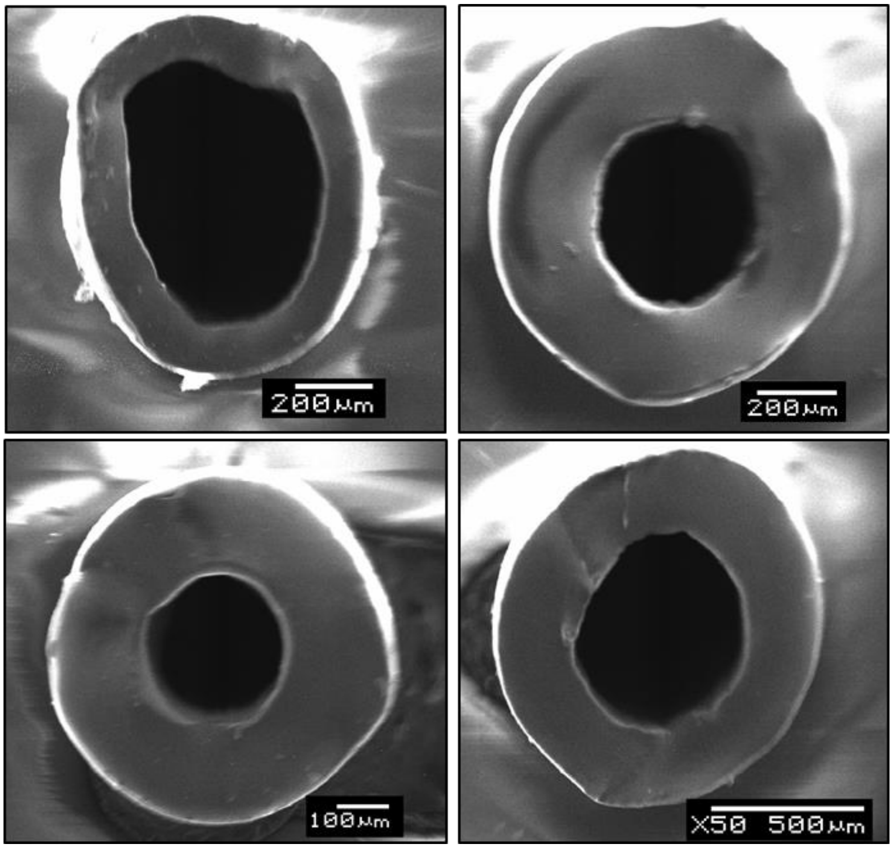

Figure S2. SEM images of the cross section for the microtubule with different inner diameter of the channel and wall thickness

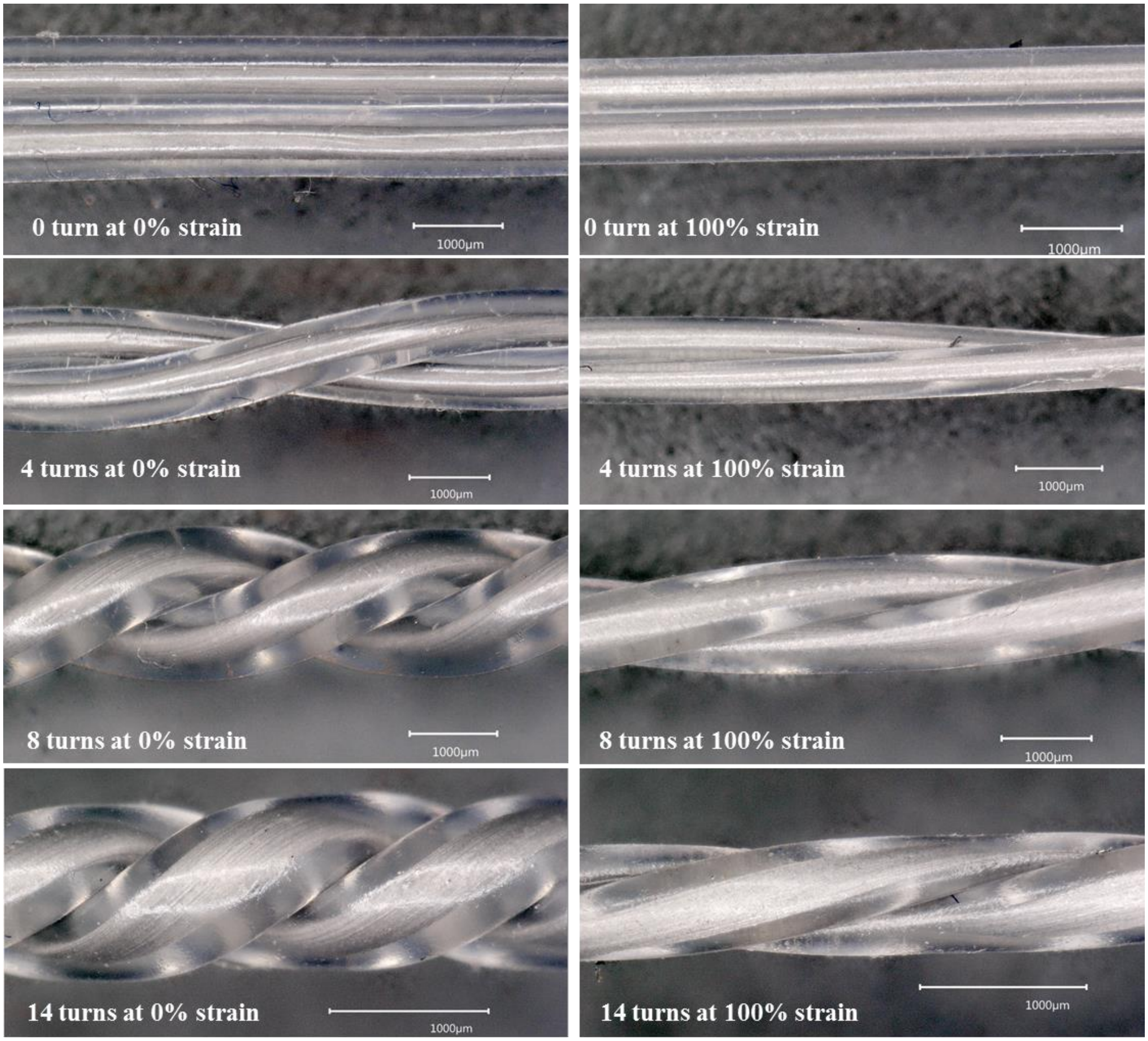

Figure S3. Optical images for twisted conductive microtubules with different strain and turns (left panel at 0% strain and right panel at 100% strain). The images are recorded using a 3D Microscope (Model VHX-5000, Keyence Corp., Japan).

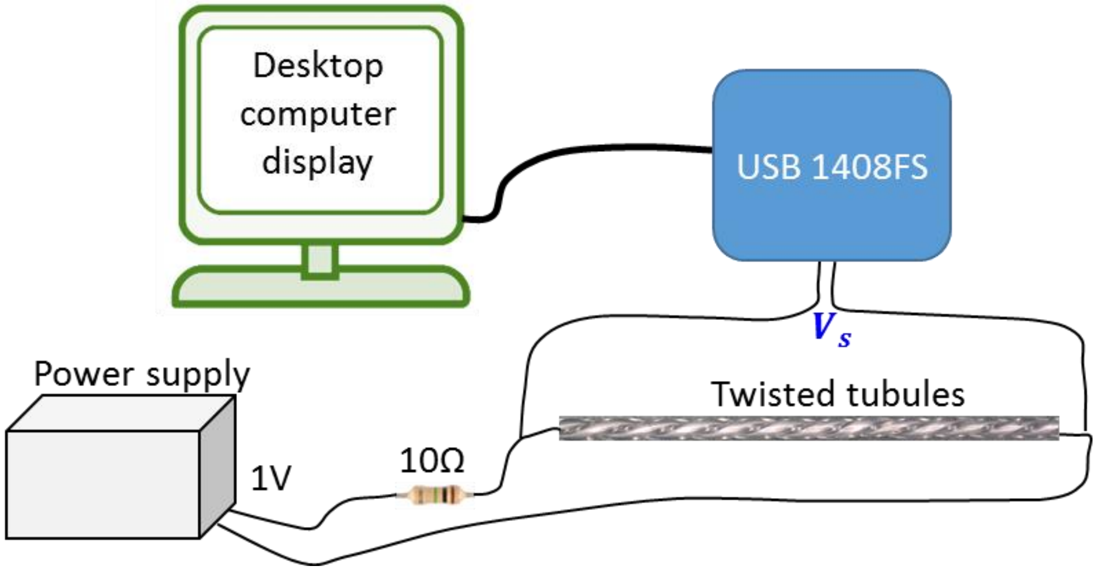

Figure S4. Indirect resistance measurement for the twisted conductive microtubules. We use a resistor of 10 Ω that directly connected to the twisted microtubules. Data acquisition hardware (USB 1408FS, MicroDAQ, ltd., USA) was connected to a Dell Desktop computer to digitize the signal. The power supply was set at 1 volt. Let V_s be the analog signal measured from both end of the sensor, the resistance R_s can be obtained as R_s=10V_s/(1-V_s).

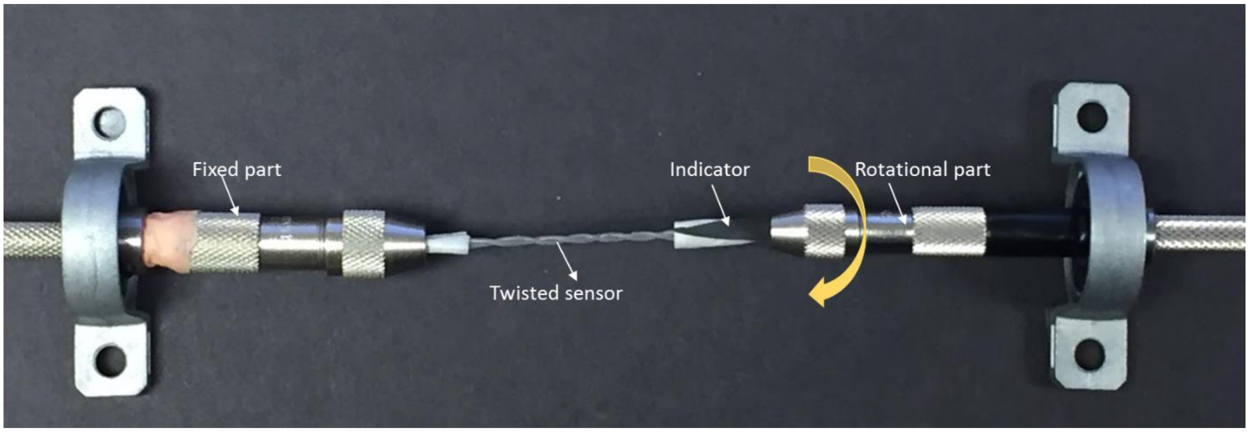

Figure S5. Conductive microtubules are twisted at desired turns using two part pin vises (Starrett, USA). One part is fixed on the base and the other is connected to a rotary bearing. The number of turns of the twisted microtubules is controlled by the number of rotational cycles from the Starrett rotational part.

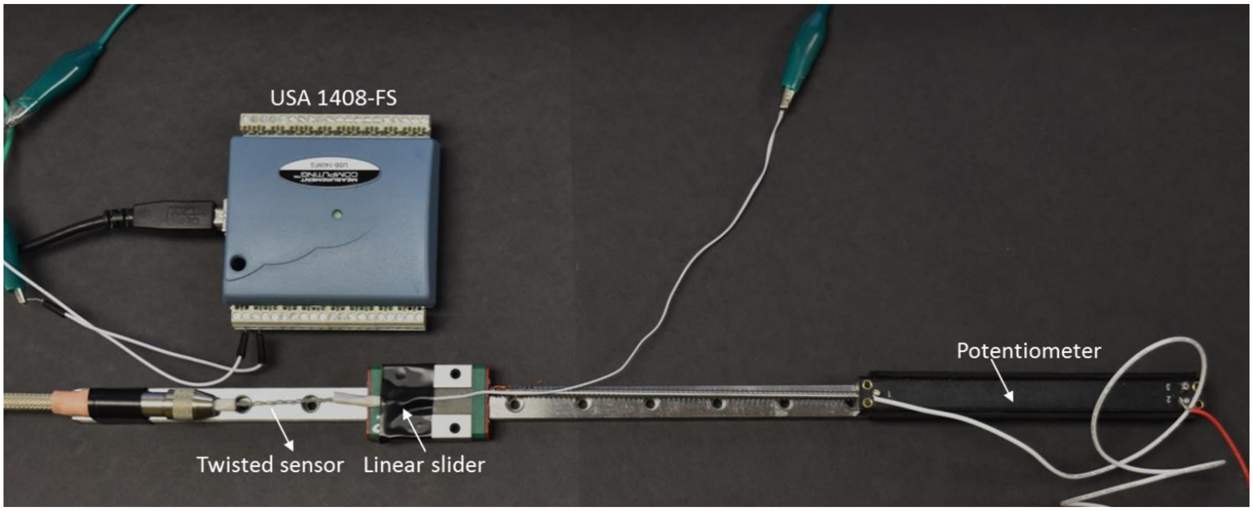

Figure S6. Strain measurement for the twisted microtubules using a potentiometer (Model PTF, State Electronics Part Corp., USA), a DAQ module (USB 1408FS, MicroDAQ, ltd., USA), and a MISUMI linear slider. The signals are captured in software (MATLAB, The MathWorks, Inc., USA).

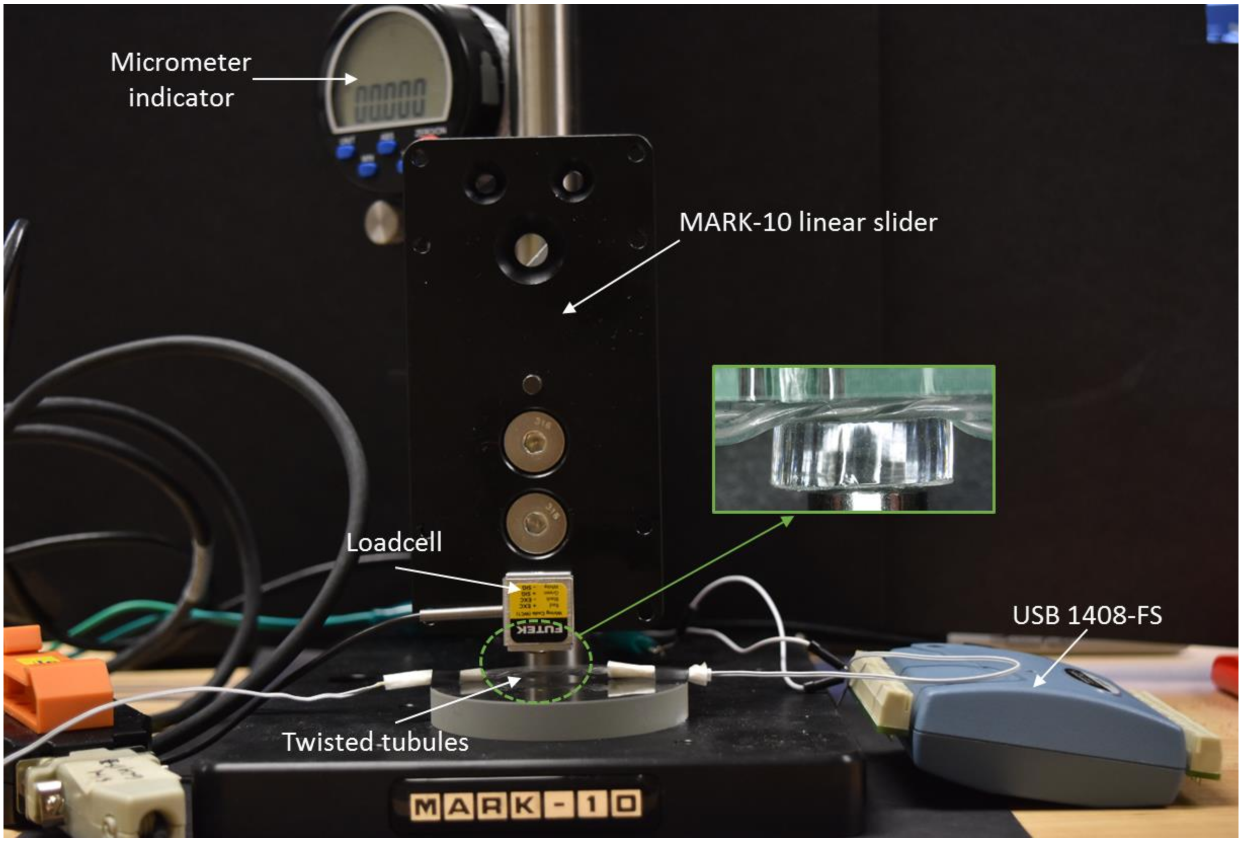

Figure S7. Experimental setup for stress-strain characterization

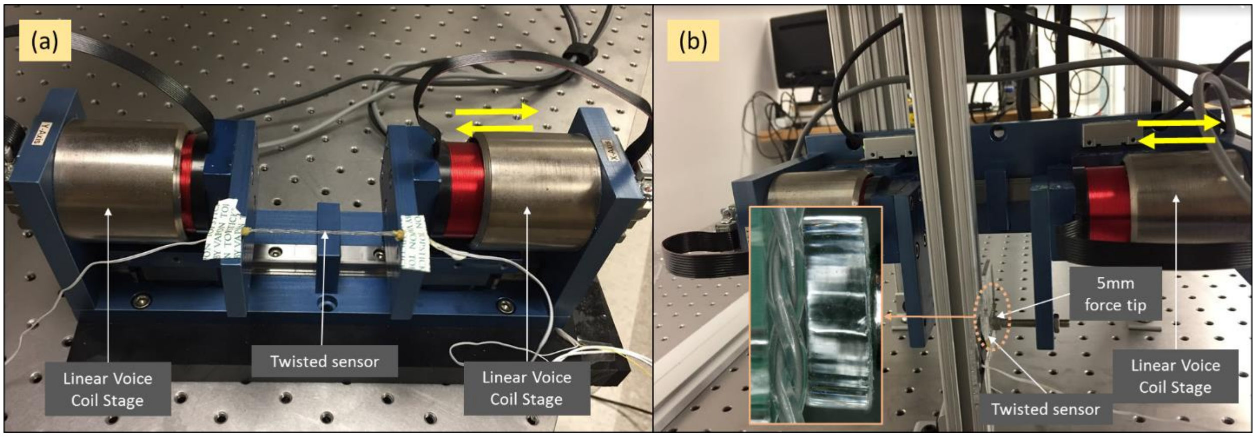

Figure S8. Strain (left panel) and stress (right panel) cycling tests for the twisted microtubules using a two-axis linear voice coil positioning stage (Model VCS15-050-LB-01-MC-2, H2W Technologies, Inc., USA).

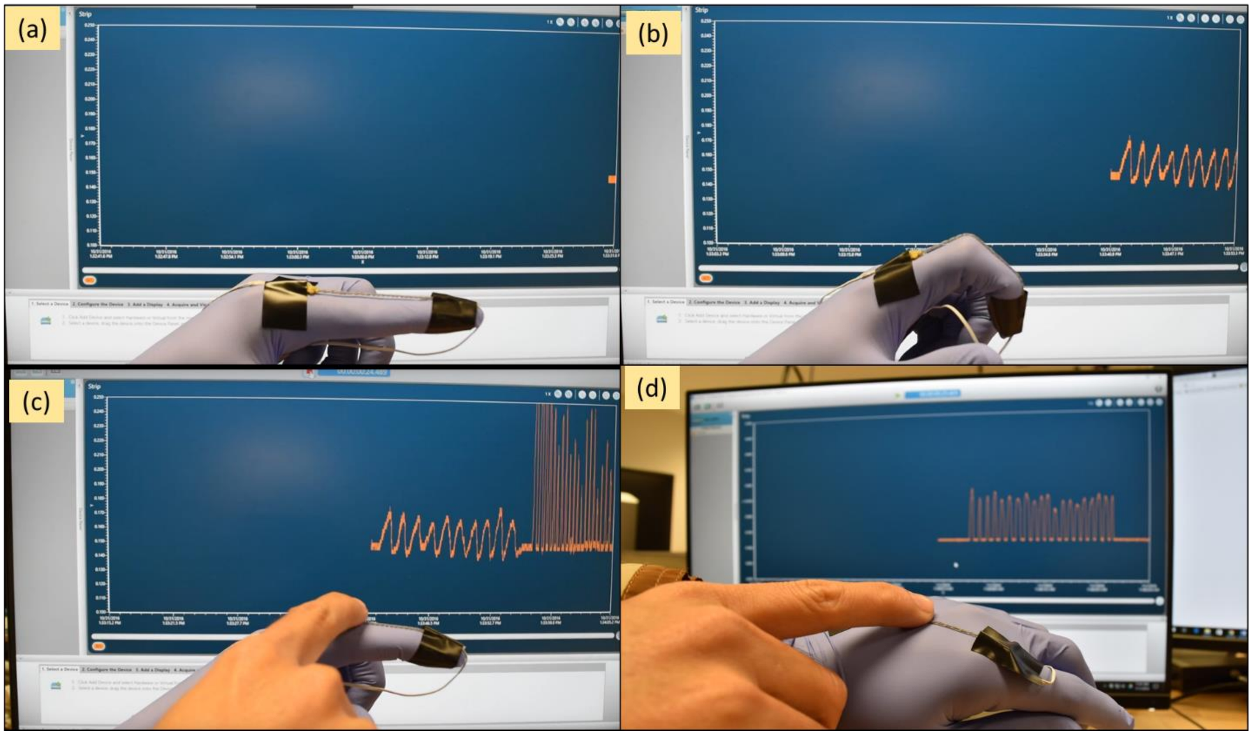

Figure S9. Optical images, bending motion sensing of the human finger using twisted microtubules with 10 turns, (a) Finger in a straight position, (b) Finger in a bending position, (c) Twisted sensor is forced by another finger, (d) Twisted sensor is mounted on the dorsal surface of left hand and under continuous load from right hand.

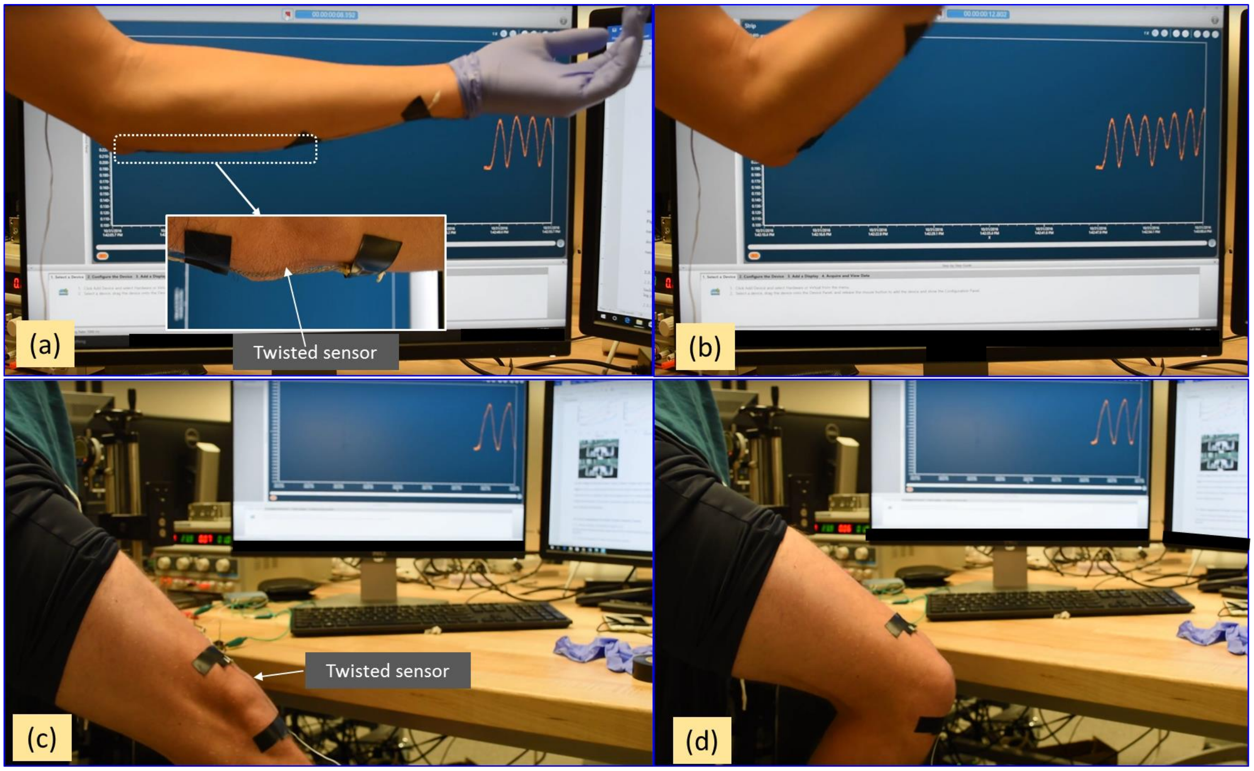

Figure S10. Optical images, bending motion sensing of the human elbow and knee joints using twisted microtubules with 10 turns, (a and b) bending motion detection for the elbow joint when the arm is in straight position and bending position, respectively, (c and d) bending motion detection for the knee joint when the leg is in straight position and bending position, respectively.

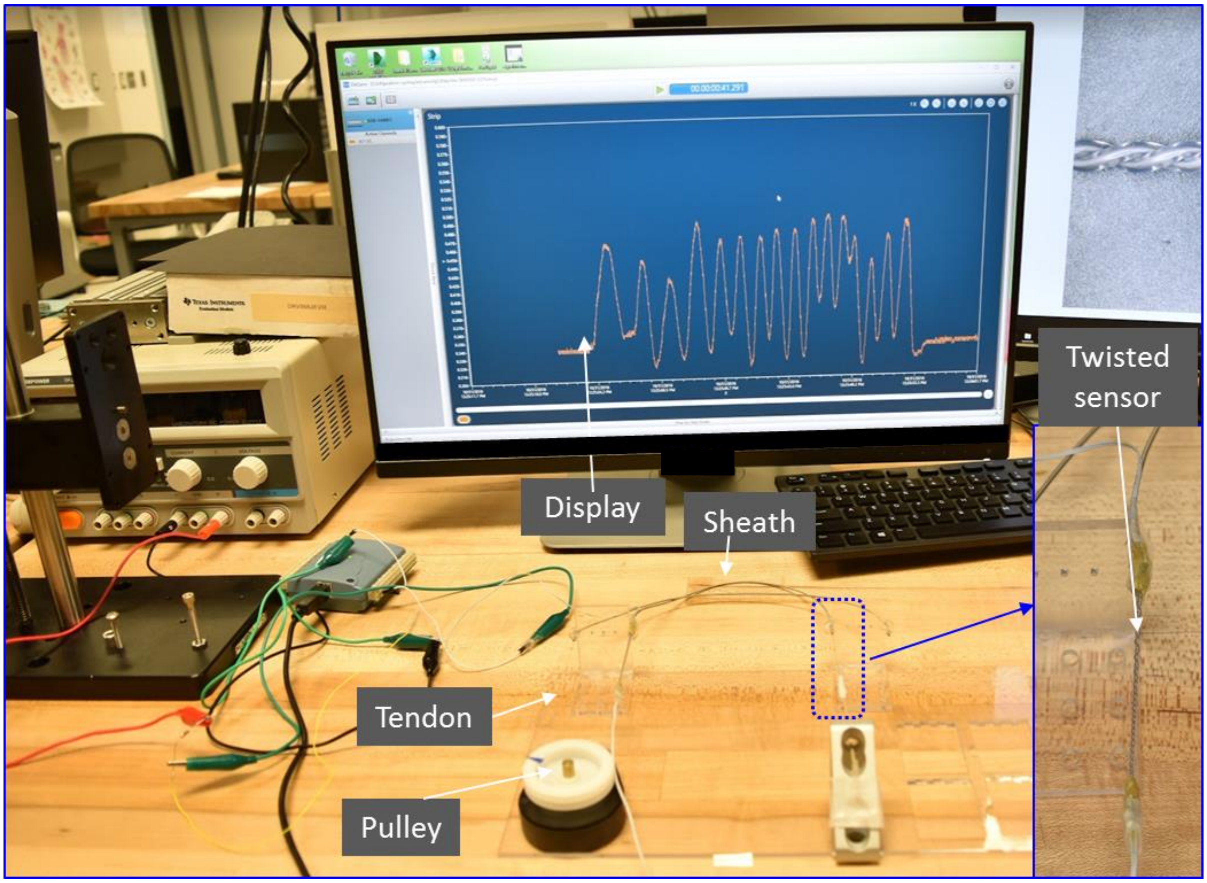

Figure S11. Experimental configuration for evaluating the twisted microtubules as position sensors integrated within a tendon-sheath mechanism-driven pulley system.

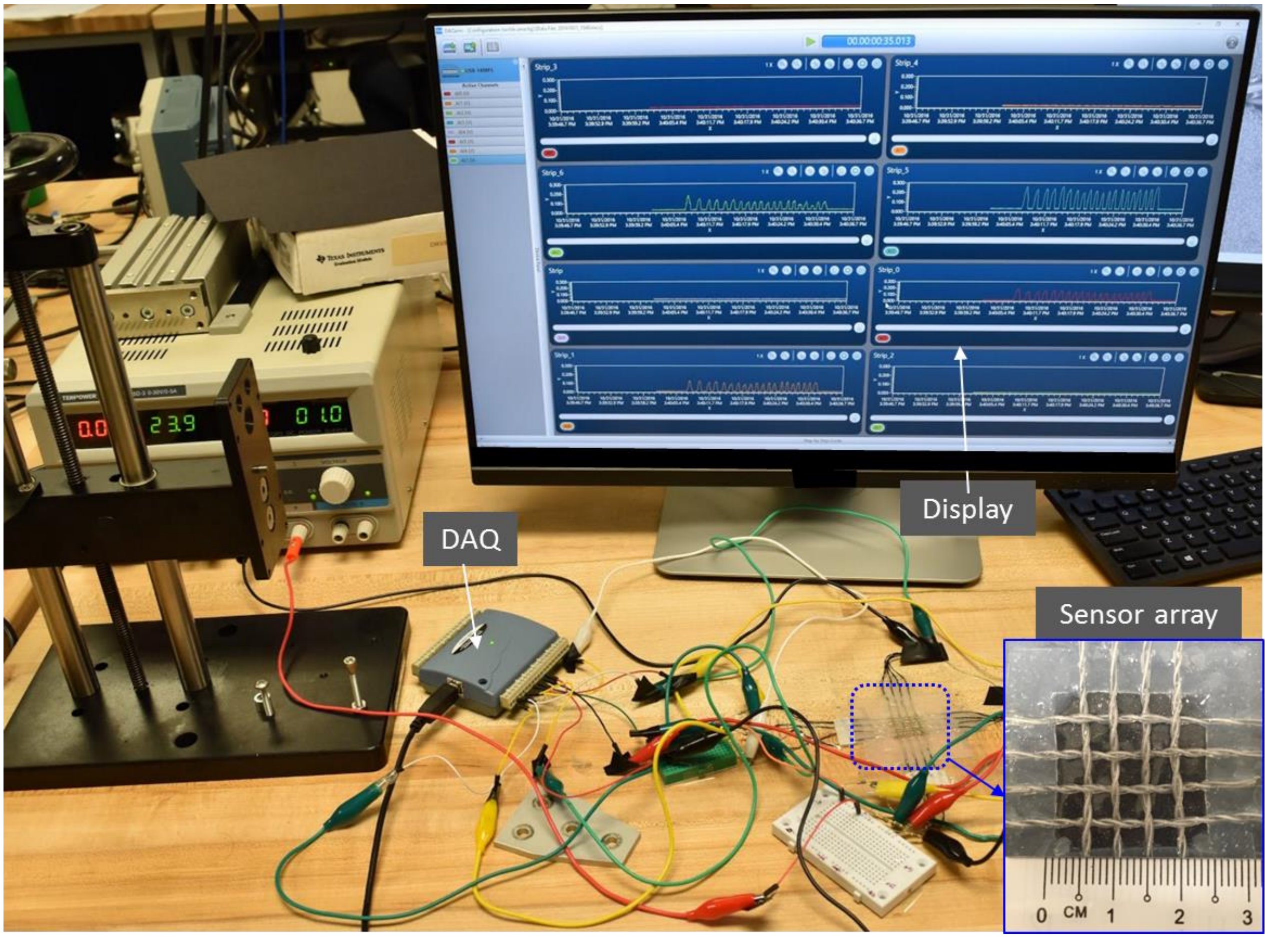

Figure S12. Experimental configuration for a tactile sensing array (4x4 sensing cells) composed of woven microtubules.

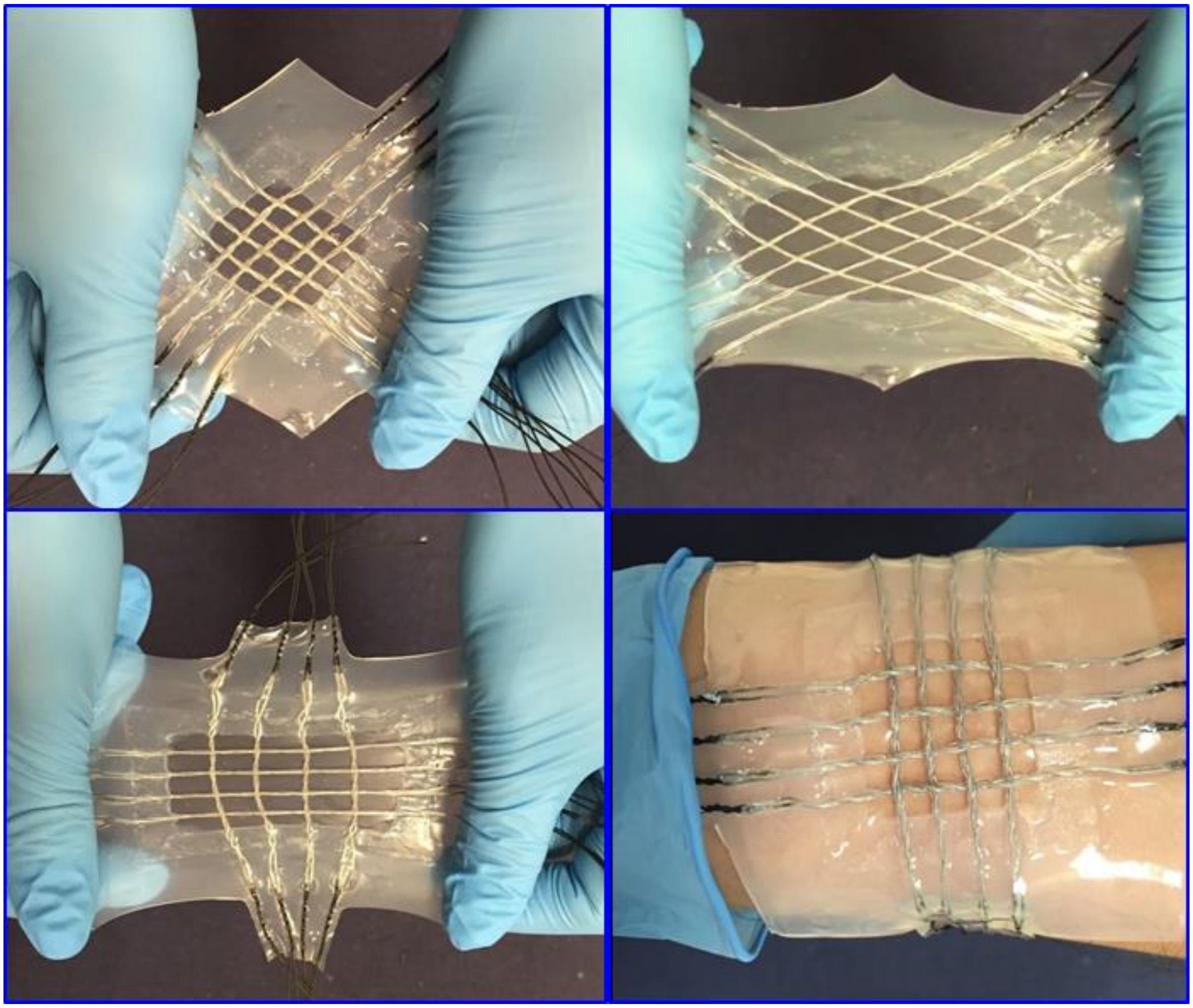

Figure S13. Mechanical stretchability for a tactile sensing array (4x4 sensing cells) worn on a human arm.

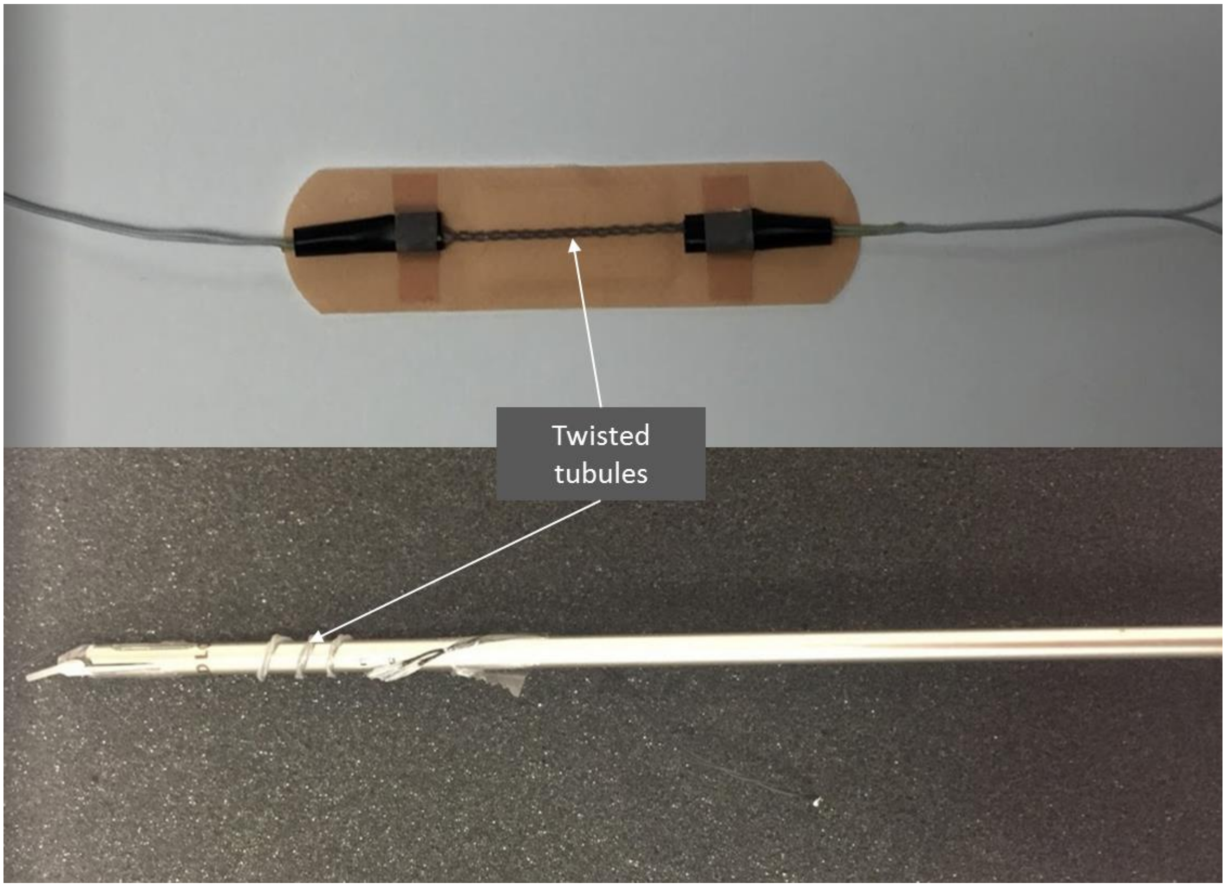

Figure S14. Integration of twisted conductive microtubules into a medical bandage (upper panel) or surgical tool (lower panel)

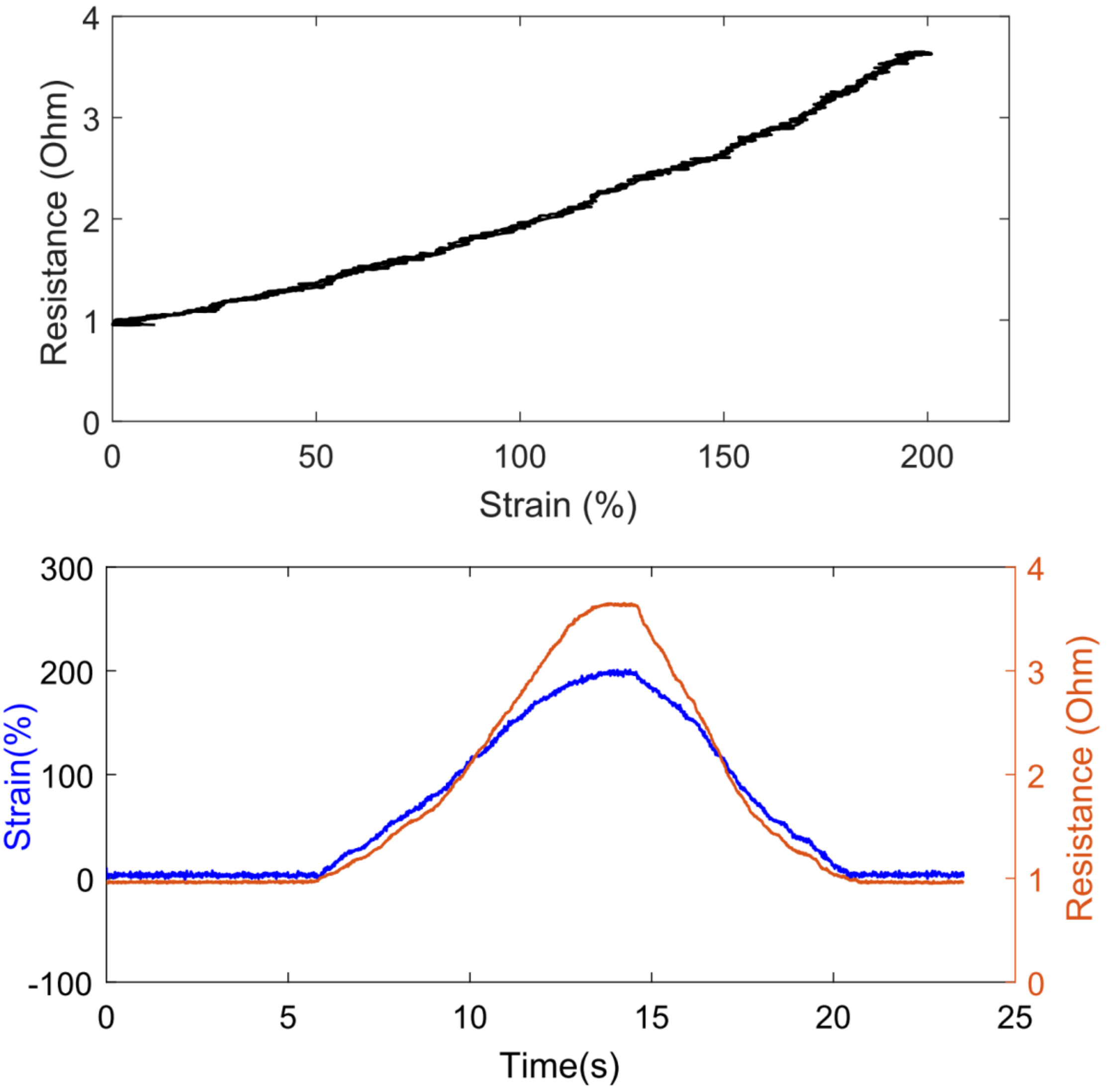

Figure S15. Loading and unloading behavior of the twisted microtubules under strain testing with 4 twisted turns. (Upper panel) Resistance versus strain, (Lower panel) Time history for the strain (left y-axis) and resistance (right y-axis).

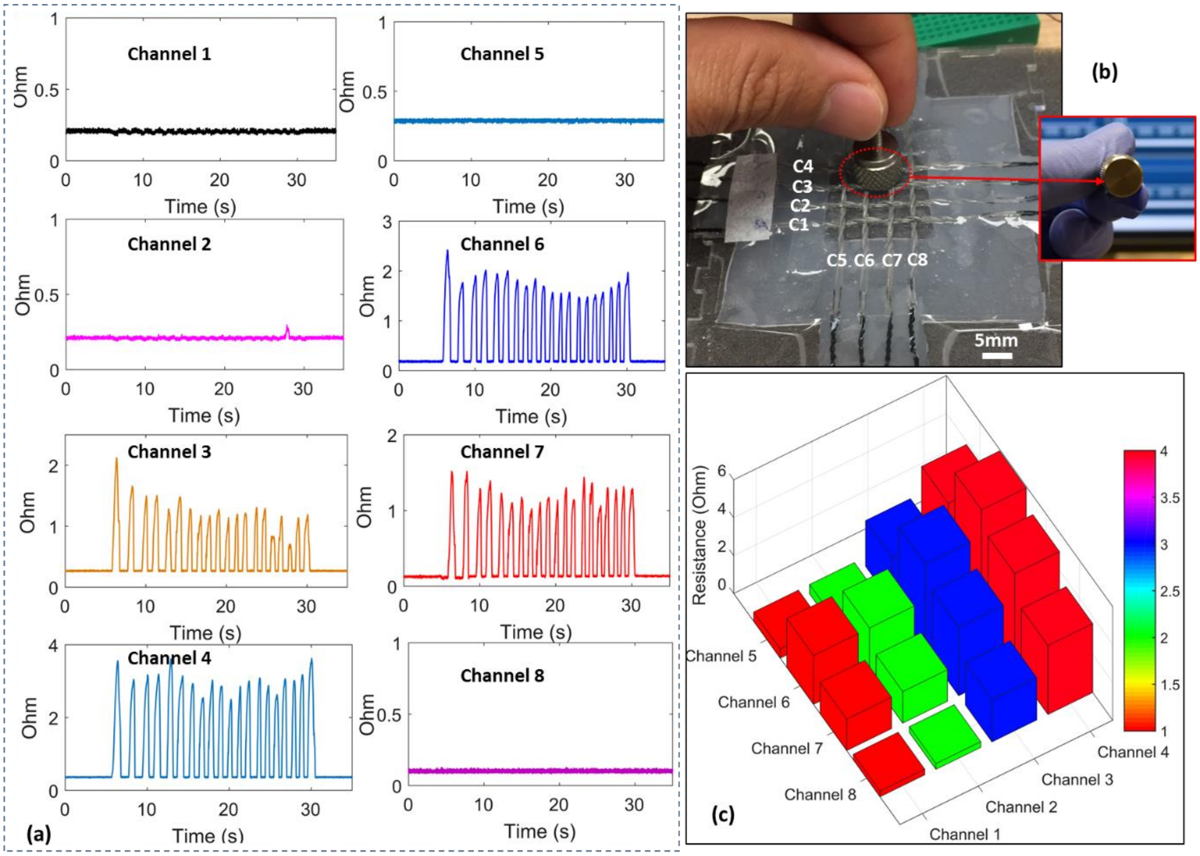

Figure S16. Woven microtubules in a tactile sensing array. (a) Contact force sensing for individual channels. (b) Optical image of tactile array (tip displacement: 10 mm). (c) The maximum force in the array correctly identifies the location at which force is applied.

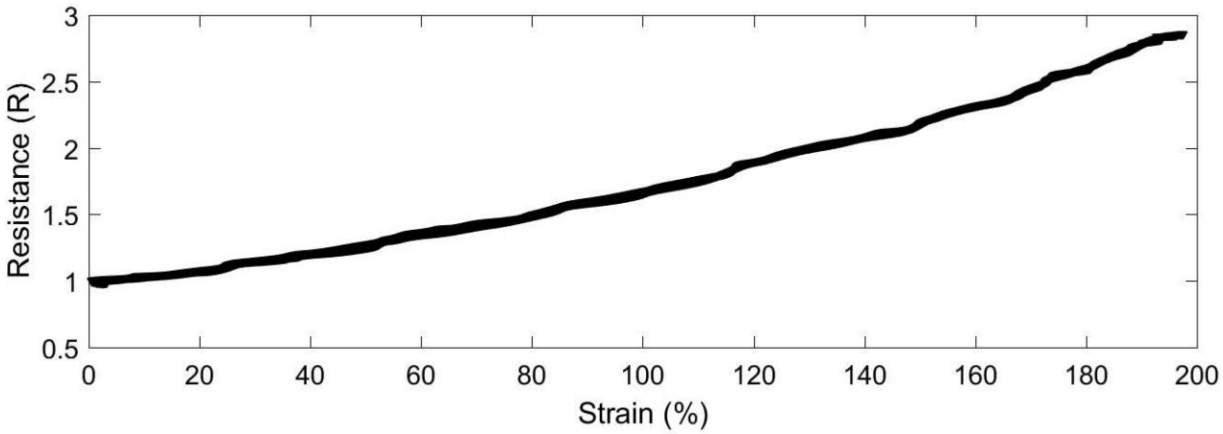

Figure S17. Strain versus resistance test result of untwisted microtubules (0 turn)

Table S1. Comparison between twisted microtubules and other conventional commercial sensors

Performance \ Sensor	**Twisted microtubules**	**Commercial conventional sensors**
Bending measurement	over 2π(rad)	over 2π(rad) [1,2]
Sharp bend	Yes	No[1,2]
Force measurement	Yes	Yes[1,2]
Stretchability	Can be stretched over 400%	can be stretched less than order of 1% [3]
Rotational measurement (Angle)	Can up to100π (rad)	Not limited [1]
Adapt with various surface and soft structure	Yes	No[4]
Sensitivity (Gauge factor-GF)	GF=1÷2	[2,5] GF is at least 1
Multifunctional capability (can perform force, position, torque sensing using a single sensor)	Yes	No[5]
Can integrate into fabric/clothes	Yes	No[5]
Soft structure	Yes	No[3-5]
Operate as conventional electrical wires	Yes	No

References

[1] Roriz, P., Carvalho, L., Frazão, O., Santos, J. L., & Simões, J. A. (2014). From conventional sensors to fibre optic sensors for strain and force measurements in biomechanics applications: A review. Journal of biomechanics, 47(6), pp. 1251-1261

[2] C. Wong, Z. Q. Zhang, B. Lo and G. Z. Yang, "Wearable Sensing for Solid Biomechanics: A Review," in IEEE Sensors Journal, vol. 15, no. 5, pp. 2747-2760, May 2015

[3] J. B. Chossat, Y. L. Park, R. J. Wood and V. Duchaine, "A Soft Strain Sensor Based on Ionic and Metal Liquids," in IEEE Sensors Journal, vol. 13, no. 9, pp. 3405-3414, Sept. 2013.

[4] Bauer, S., Bauer-Gogonea, S., Graz, I., Kaltenbrunner, M., Keplinger, C. and Schwödiauer, R. (2014), 25th Anniversary Article: A Soft Future: From Robots and Sensor Skin to Energy Harvesters. Adv. Mater., 26: 149–162

[5] Amjadi, M., Kyung, K.-U., Park, I. and Sitti, M. (2016), Stretchable, Skin-Mountable, and Wearable Strain Sensors and Their Potential Applications: A Review. Adv. Funct. Mater., 26: 1678–1698
